# SMAD4 Feedback Activates the Canonical TGF-β Family Signaling Pathways

**DOI:** 10.3390/ijms221810024

**Published:** 2021-09-16

**Authors:** Lu Liu, Qiqi Li, Liu Yang, Qifa Li, Xing Du

**Affiliations:** College of Animal Science and Technology, Nanjing Agricultural University, Nanjing 210095, China; 2017105015@njau.edu.cn (L.L.); 2018205004@njau.edu.cn (Q.L.); 2019205011@njau.edu.cn (L.Y.); liqifa@njau.edu.cn (Q.L.)

**Keywords:** SMAD4, TGF-β family signaling pathways, feedback regulation, pig, granulosa cells, transcriptome, RNA sequencing

## Abstract

TGF-β family signaling pathways, including TGF-β and BMP pathways, are widely involved in the regulation of health and diseases through downstream SMADs, which are also regulated by multiple validated mechanisms, such as genetic regulation, epigenetic regulation, and feedback regulation. However, it is still unclear whether R-SMADs or Co-SMAD can feedback regulate the TGF-β family signaling pathways in granulosa cells (GCs). In this study, we report a novel mechanism underlying the feedback regulation of TGF-β family signaling pathways, i.e., SMAD4, the only Co-SMAD, positive feedback activates the TGF-β family signaling pathways in GCs with a basal level of TGF-β ligands by interacting with the core promoters of its upstream receptors. Mechanistically, SMAD4 acts as a transcription factor, and feedback activates the transcription of its upstream receptors, including *ACVR1B*, *BMPR2*, and *TGFBR2*, of the canonical TGF-β signaling pathways by interacting with three coactivators (c-JUN, CREB1, and SP1), respectively. Notably, three different interaction modes between SMAD4 and coactivators were identified in SMAD4-mediated feedback regulation of upstream receptors through reciprocal ChIP assays. Our findings in the present study indicate for the first time that SMAD4 feedback activates the canonical TGF-β family signaling pathways in GCs, which improves and expands the regulatory mechanism, especially the feedback regulation modes of TGF-β family signaling pathways in ovarian GCs.

## 1. Introduction

The transforming growth factor-β (TGF-β) superfamily is a large group of phylogenetically conserved secreted cytokines in eukaryotes, which contains more than 30 members, including TGF-βs, activins, inhibins, bone morphogenetic proteins (BMPs), and growth and differentiation factors (GDFs) [1]. Members of the TGF-β superfamily are widely expressed in various tissues and cells, which play critical roles in the regulation of multiple crucial biological processes associated with health and disease, mainly by activating or inhibiting the TGF-β family signaling pathways [2]. In general, the TGF-β family signaling pathway can be divided into two branches, i.e., TGF-β and BMP signaling pathways, which both signal in the order of ligands, receptors (including type II and type I receptors), receptor-regulated SMADs (R-SMADs: SMAD2/3 for the TGF-β signaling pathway, and SMAD1/5/8 for the BMP signaling pathway), and the only common mediator SMAD (Co-SMAD: SMAD4), which finally shuttles into the nucleus to regulate the transcription of target genes [3].

TGF-β family signaling pathways have been shown to be regulated by multiple validated mechanisms at different levels, such as genetic regulation, epigenetic regulation, and feedback regulation [4,5]. Feedback regulation is nowadays identified and defined as downstream signaling proteins that regulate the expression or activity of upstream signaling molecules directly or indirectly (via other regulators or signaling axis), which is also known as the main feedback model of TGF-β family signaling pathways [6]. The regulators involved in this feedback regulation progresses includes (i) miRNAs, such as SMAD4-miR-675-TGFBR1 [6], SMAD4-miR-425-TGFBR2 [7], SMAD4-miR-302-BMPR2 [8], and SMAD4-miR-24-3p-SMAD2 [9]; (ii) lncRNAs, such as SMAD2/3-lncRNA-MALAT1-TGFBR2 [10]; (iii) transcription factors (TFs), such as β-catenin (mediates SMAD4 induction of receptor) [11]; and (iv) transcriptional coregulators, such as Snail [12]. Additionally, the experimentally validated axes, including the SCF/STAT3 axis (mediates SMAD2 positive feedback regulation of TGF-β1) [13], SLIT2-Gremlin axis (mediates SMAD1/5/8/4 complex regulation of BMP2) [14], and so on. In addition, TGF-β and BMP signals also form feedback loops in a regulator-dependent manner, such as PML/PIN1 for TGF-β1 [15], NCX1/TRPC6 complex for SMAD2 [16], lncRNA-Crnde for Smad3 [17], DSPP/DSP for SMAD1/5/8 [18], and HNF4 for SMAD4 [19] to feedback regulate the expression of itself.

Unlike R-SMADs and Co-SMAD, inhibitory SMADs (SMAD6 and SMAD7), especially SMAD7, feedback against the TGF-β family signaling pathways by directly interacting with the members of TGF-β family signaling pathway at the protein [20] or gene (mainly promoter region) level [21]. However, it is still unknown whether R-SMADs or Co-SMAD can directly feedback regulate the TGF-β family signaling pathway. One of the main reasons for the unclear situation is the lack of investigation for their effects on the intracellular transcriptomic alteration and without screening their potential target genes. Here, we focused on characterizing SMAD4-mediated transcriptomic alteration and expected to identify the potential functional targets of SMAD4 in porcine GCs due to the irreplaceable roles of SMAD4 in maintaining ovary development, as well as the normal states and functions of GCs.

In the present study, we reanalyzed global transcriptional alteration in porcine GCs after SMAD4 knockdown and identified functional differentially expressed mRNAs (DEmRNAs). Among the downregulated DEmRNAs, *ACVR1B*, *BMPR2*, and *TGFBR2*, three upstream TGF-β family receptors of SMAD4 were selected for further research. Interestingly, our findings show that *ACVR1B*, *BMPR2*, and *TGFBR2* are feedback induced by SMAD4 in porcine GCs with a basal level of TGF-β ligands. Intriguingly, SMAD4, the only Co-SMAD, acts as a TF and positive feedback activates the TGF-β family signaling pathway in porcine GCs by binding to the promoter region of its upstream receptor genes (*ACVR1B*, *BMPR2*, and *TGFBR2*), which is mechanically dependent on three transcriptional coactivators (c-JUN, CREB1, and SP1) with different interaction modes.

## 2. Results

### 2.1. Reanalysis of the Transcriptomic Alteration in Porcine GCs after Knockdown of SMAD4

**Figure 1:**
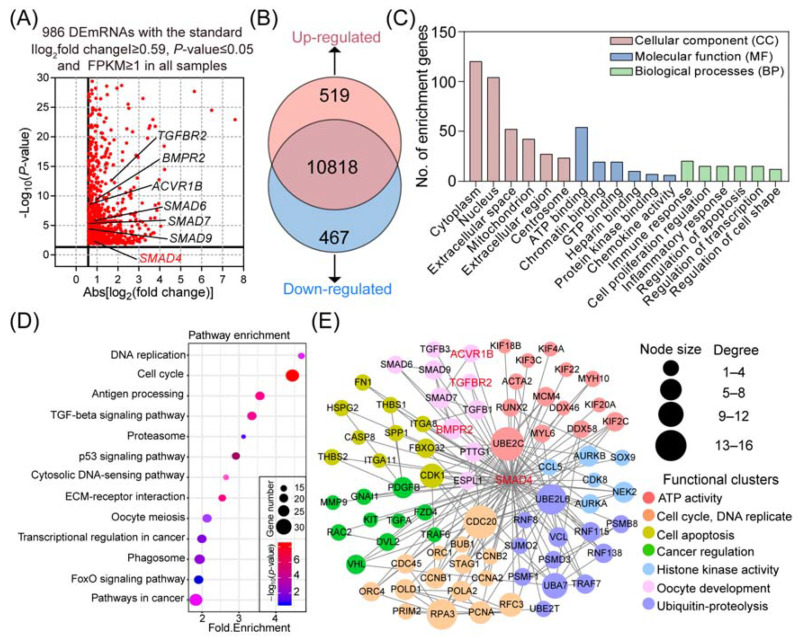
Reanalysis of the transcriptomic alteration in porcine GCs after SMAD4 knockdown. (**A**) Volcano plot of the identified DEmiRNAs. The DEmRNAs in porcine GCs treated with SMAD4 siRNA are shown as red dots. *x*-axis and *y*-axis are described according to |log_2_(fold change)| and −log_10_(*p*-value), respectively. (**B**) Expression pattern distribution of DEmRNAs under SMAD4-silencing. (**C**) Gene Ontology (GO) analyses of the DEmRNAs in porcine GCs after knockdown of SMAD4. The top 6 significantly enriched function terms in CC, MF, and BP categories were represented. (**D**) KEGG pathway analyses of the DEmRNAs in porcine GCs under SMAD4 knockdown and 13 significantly enriched pathways were presented. (**E**) Identification of the SMAD4-mediated gene–function network. The function-known DEmRNAs are shown as circles (nodes). Edges indicate the potential interaction between different nodes. The size of nodes reflected their degree in the network. Nodes are clustered according to the biological functions as indicated.

In this study, with the background of *Sus Scrofa* RefSeq 11.1 (*Sscrofa* 11.1), we reanalyzed our previous RNA-seq data (GSE65696) obtained from porcine GCs after knockdown of SMAD4, which was originally mapped to the *Sscorfa* RefSeq 10.2 [22]. After examination, a total of 11,804 genes were mapped, and 986 DEmRNAs were identified with the criteria |−log_2_(fold change)| ≥ 0.59, *p*-value ≤ 0.05, and FPKM ≥ 1 in all samples (Figure 1A). Among them, 519 DEmRNAs were significantly upregulated, and the other 467 DEmRNAs were dramatically downregulated (Figure 1B). All the DEmRNAs identified here are presented in Table S1.

To further explore the potential biological functions and physiological processes associated with the SMAD4-induced DEmRNAs, Gene Ontology (GO) and KEGG pathway enrichment analyses were performed. As shown in Table S2, a total of 83 significantly enriched GO terms were identified in three categories, including 18 (21.7%) in cellular components (CC), 53 (63.9%) in biological processes (BP), and 12 (14.4%) in molecular function (MF). Functional analyses showed that these identified DEmRNAs are mainly associated with functional groups, which are essential for transcription regulation, such as chromatin binding, protein kinase binding, and gene expression regulation (Figure 1C). Furthermore, the results obtained from KEGG pathway analyses showed that the SMAD4-induced DEmRNAs were significantly enriched in 13 crucial pathways involved in the regulation of cell states, functions, and stimulation response, such as the TGF-β, FOXO, and p53 signaling pathways (Figure 1D, Table S3).

To establish the SMAD4-mediated gene–function interaction network, the identified DEmRNAs were analyzed by the STRING protein–protein interaction (PPI) database and clustered based on their known functions obtained from GO analysis (Figure S1). Among them, a highly interacted network with 78 DEmRNAs and 171 interactions was noticed and selected for further research (Figure 1E). After functional assessment, seven major subclusters were identified within the network, including cell apoptosis, cycle, and oocyte development, which provides a systematic view of the multiple biological functions of SMAD4 in diverse biological processes.

### 2.2. SMAD4 Is a Strong Inducer for Its Upstream Receptors

**Figure 2:**
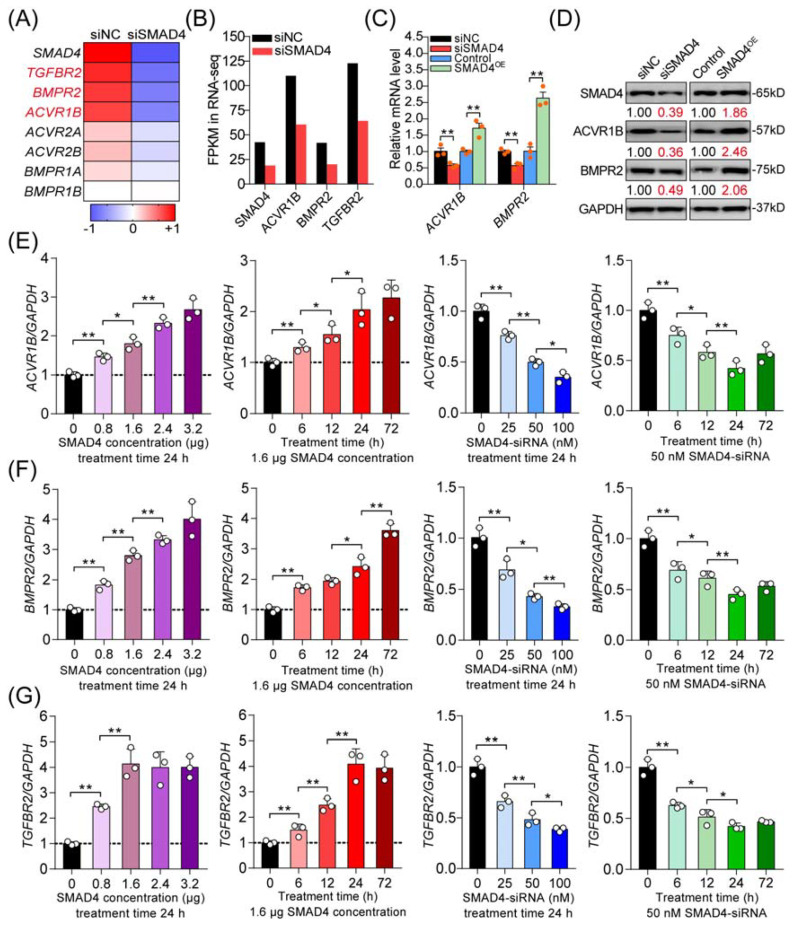
SMAD4 feedback induces the transcription of its upstream receptor genes (*ACVR1B*, *BMPR2*, and *TGFBR2*) in porcine GCs. (**A**) Heat map showing the expression pattern of TGF-β family signaling receptors in porcine GCs after SMAD4 silencing (siSMAD4). The color scale indicates the expression degree, increase (red), and decrease (blue). (**B**) Expression levels (FPKM) of *SMAD4*, *ACVR1B*, *BMPR2*, and *TGFBR2* in SMAD4-silenced porcine GCs according to RNA-seq. (**C**,**D**) The mRNA (**C**) and protein (**D**) levels of ACVR1B and BMPR2 in SMAD4-expressed or -inhibited porcine GCs were determined by qRT-PCR and Western blotting assays (*n* = 3). (**E**–**G**) The mRNA levels of *ACVR1B* (**E**), *BMPR2* (**F**), and *TGFBR2* (**G**) in porcine GCs treated with pcDNA3.1-SMAD4 or SMAD4-siRNA with different concentrations and treatment times as indicated were analyzed by qRT-PCR (*n* = 3). The data in (**C**,**E**–**G**) were normalized by *GAPDH* and are shown as the mean ± S.D. of three independent experiments. *p*-values were calculated by a two-tailed Student’s *t*-test, * *p* < 0.05, ** *p* < 0.01.

Next, we analyzed the differentially expressed unigenes in porcine GCs after SMAD4 knockdown and, interestingly, noticed that six receptors of the TGF-β family signaling pathways were downregulated (Figure 2A). Among them, three receptors also identified in Figure 1E, including ACVR1B (the canonical activin type I receptor), BMPR2 (type II receptor for BMPs), and TGFBR2 (type II receptor for TGF-βs), were significantly downregulated in porcine GCs treated with SMAD4-siRNA (Figure 2B), which were chosen for further research. Our previous study demonstrated that SMAD4 could feedback enhances *TGFBR2* expression through miR-425 [7]. To further evaluate the identification results, we examined the effects of SMAD4 on the expression levels of *ACVR1B* and *BMPR2* in porcine GCs. With the results obtained from gain or loss of function, we confirmed that SMAD4 could positive-feedback regulate the expression of *ACVR1B* and *BMPR2*, the other two receptors of TGF-β family signaling pathways, at both mRNA and protein levels in porcine GCs (Figure 2C,D). Furthermore, we analyzed the expression levels of *ACVR1B*, *BMPR2*, and *TGFBR2* in porcine GCs treated with pcDNA3.1-SMAD4 or SMAD4-siRNA with different concentrations and times as indicated. The results demonstrated that overexpression of SMAD4 significantly induced, but knockdown of SMAD4 dramatically inhibited, the mRNA levels of *ACVR1B*, *BMPR2*, and *TGFBR2* in a dose- and time-dependent manner (Figure 2E–G and Figure S2). Taken together, the results demonstrate that SMAD4 is a strong inducer for the transcription of its upstream receptors (*ACVR1B*, *BMPR2*, and *TGFBR2*), which further indicates the feedback activity of the SMAD4 to TGF-β family signaling pathways in porcine GCs with a basal level of TGF-β ligands.

### 2.3. SMAD4 Feedback Regulates Its Upstream Receptors by Acting as a Transcription Factor

**Figure 3:**
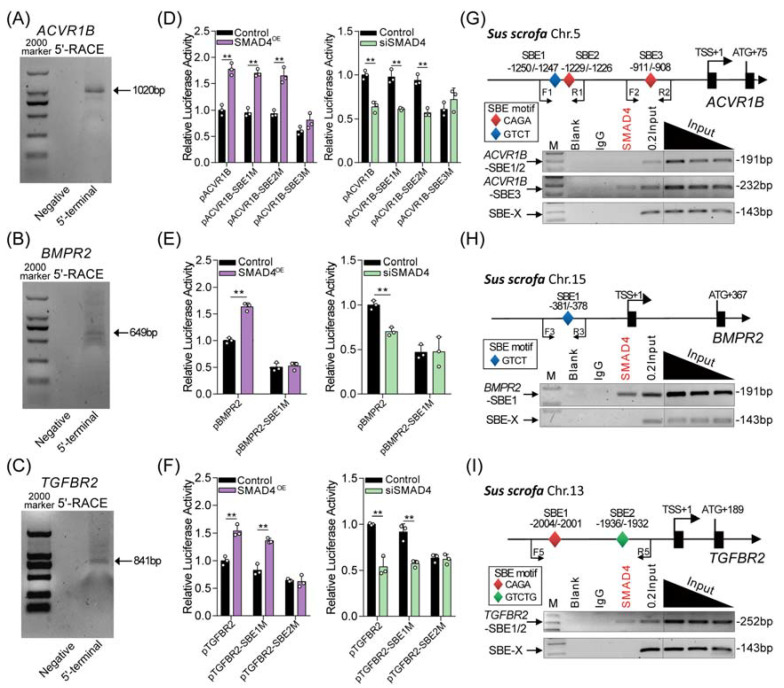
SMAD4 acts as a TF and binds to the core promoter of pig *ACVR1B*, *BMPR2*, and *TGFBR2***.** (**A**–**C**) Identification of the transcription start sites (TSS) of pig *ACVR1B*, *BMPR2*, and *TGFBR2* by RACE assay. Gel images depicting the 5′-RACE amplification products of pig *ACVR1B* (**A**), *BMPR2* (**B**) and *TGFBR2* (**C**). 5′-terminal indicates the gene-specific primer (GSP) addition and negative means no GSP primer. The size of each band is indicated by an arrow. (**D**–**F**) The effects of SMAD4 on the promoter activity of pig *ACVR1B* (**D**), *BMPR2* (**E**) and *TGFBR2* (**F**) with wild-type or mutant-type SBEs were detected by luciferase activity assays (*n* = 3). The location of different SBE motifs within the promoter of pig *ACVR1B*, *BMPR2*, and *TGFBR2* were analyzed and shown in Figure S3 and (**G**–**I**). (**G**–**I**) Potential binding sites of SMAD4 within the core promoter of pig *ACVR1B* (**G**), *BMPR2* (**H**), and *TGFBR2* (**I**) were identified by ChIP assays. Different SBE motifs were indicated by diamonds with different colors. Specifically, red, blue, and green diamonds indicate “CAGA”, “GTCT”, and “GTCTG” motifs, respectively. SBE-X indicates the negative control for ChIP assays. The data in (**D**–**F**) are shown as the mean ± S.D. with three independent experiments. *p*-values were calculated by a two-tailed Student’s *t*-test, ** *p* < 0.01.

It has been proven that SMAD4 regulates target gene expression by acting as a transcription factor. We therefore speculated that SMAD4 feedback induces the transcription of upstream receptors (*ACVR1B*, *BMPR2*, and *TGFBR2*) with its transcription factor activity. To address this, the transcription start site (TSS) of pig *ACVR1B*, *BMPR2*, and *TGFBR2* gene was first identified by RACE assays (Figure 3A–C). Then, their core promoters were identified by using bioinformatics analysis and the dual-luciferase reporter system. As shown in Figure S3, the core promoter of *ACVR1B*, *BMPR2*, and *TGFBR2* was located at -1236/-952, -487/-195, and -2128/-1890 (TSS was considered +1), respectively. After analysis, several SMAD4-binding elements (SBEs) were predicted, located within the core promoter region of pig *ACVR1B* (*n* = 3), *BMPR2* (*n* = 1), and *TGFBR2* (*n* = 2) (Figure S4), suggesting that SMAD4 may interact with the core promoters of its upstream receptors and further regulate their transcription. To determine this, reporter vectors containing the core promoter of pig *ACVR1B*, *BMPR2*, and *TGFBR2* with wild-type or mutant-type SBEs were constructed and then cotransfected with pcDNA3.1-SMAD4 or SMAD4-siRNA into porcine GCs. After 24 h, luciferase activity assays were performed and showed that overexpression of SMAD4 (SMAD4^OE^) significantly increased, but knockdown of SMAD4 (siSMAD4) decreased the activity of wild-type reporters and had an effect on the activity of reporters containing *ACVR1B* promoter with SBE3 mutation, *BMPR2* promoter with SBE1 mutation, and *TGFBR2* promoter with SBE2 mutation when compared to that in the control group (Figure 3D–F and Figure S3). Additionally, we also noticed that SBE3 mutation in the *ACVR1B* promoter, SBE1 mutation in the *BMPR2* promoter, and SBE2 mutation in the *TGFBR2* promoter dramatically inhibited the transcription activity compared to each wild-type reporter, respectively. In addition, chromatin immunoprecipitation (ChIP) assays were performed and showed that SMAD4 interacts with the core promoters of pig *ACVR1B*, *BMPR2*, and *TGFBR2* genes by recognizing the corresponding SBE (Figure 3G–I). These observations above suggest that SMAD4 functions as a transcription factor, and positive-feedback regulates the transcription of its upstream receptor genes (*ACVR1B*, *BMPR2*, and *TGFBR2*) by binding to their core promoters in GCs with a basal level of TGF-β ligands.

### 2.4. Three Coactivators (CREB1, c-JUN, and SP1) Are Essential for SMAD4-Mediated Feedback Regulation of TGF-β Family Signaling Pathways

**Figure 4:**
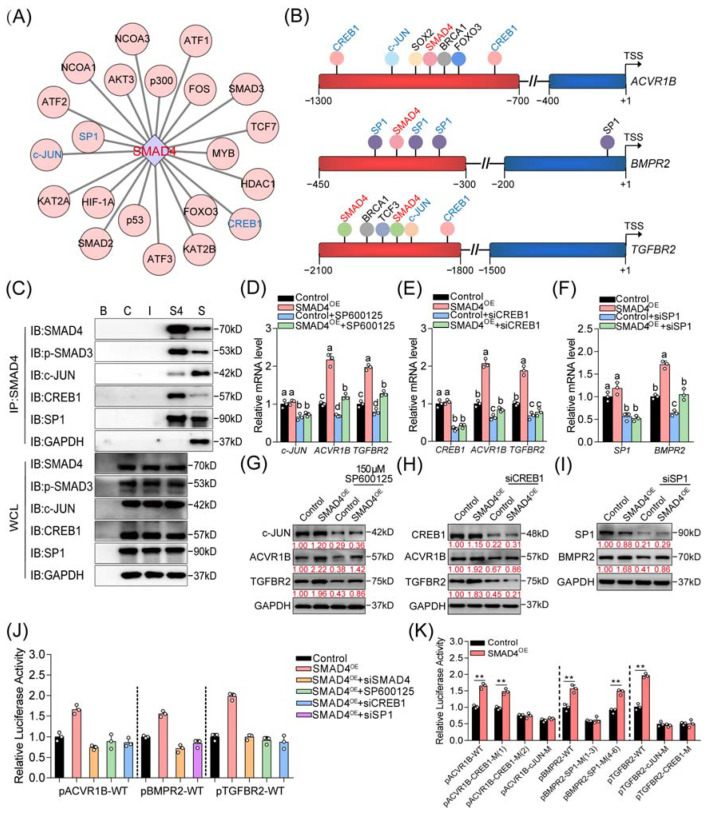
CREB1, c-JUN, and SP1 are essential coactivators for SMAD4 feedback regulation of its upstream receptors. (**A**) Identification of the SMAD4-interacted protein in mammalian GCs. The interactions between SMAD4 and 21 different TFs or coregulators in mammals were obtained from the STRING v11.0 database and visualized by Cytoscape v3.7.2 software. (**B**) Diagram showing the location of the binding motifs of candidate transcriptional coregulators within the core promoter of pig *ACVR1B*, *BMPR2*, and *TGFBR2*. The core promoter of each gene is shown as a red column. The SBEs identified in this study are labeled in red font, and the binding motifs of c-JUN, CREB1, and SP1 are indicated in blue font. (**C**) The physical interactions between SMAD4 and candidate coregulators (c-JUN, CREB1, and SP1) in porcine GCs were identified using IP assay. B, blank group; C, control group; I, IgG group; S4, anti-SMAD4 group; S, supernatant. WCL indicates the whole cell lysis which was used as positive control. (**D**–**I**) Porcine GCs were treated with 150 μM of SP600125 (**D**,**G**), siCREB1 (**E**,**H**), or siSP1 (**F**,**I**) in the absence or presence of SMAD4 stimulation for 24 h and 48 h, and the mRNA and protein levels of candidate coregulators (c-JUN, CREB1, and SP1) and SMAD4 upstream receptors (ACVR1B, BMPR2, and TGFBR2) in porcine GCs were detected by qRT-PCR and Western blotting assays, respectively (*n* = 3). (**J**) The effects of coactivators knockdown on the promoter activities of *ACVR1B*, *BMPR2*, and *TGFBR2* in SMAD4-overexpressed porcine GCs were detected by luciferase activity assays (*n* = 3). (**K**) The effects of SMAD4 overexpression on the activity of reporters containing *ACVR1B*, *BMPR2*, and *TGFBR2* promoter with the wild-type or mutant-type binding elements of CREB1, SP1, and c-JUN were detected by luciferase activity assay (*n* = 3). The location of c-JUN, CREB1, and SP1 binding motifs within the promoter of pig *ACVR1B*, *BMPR2*, and *TGFBR2* were analyzed by JASPAR and GCBI database, as shown in Figure S5. The data in (**D**–**K**) are shown as the mean ± S.D. of three independent experiments. *p*-values in (**K**) were calculated by a two-tailed Student’s *t*-test, ** *p* < 0.01. a–d, different letters in (**D**–**F**,**J**) indicate the significant differences (*p* < 0.05) among different treatment groups, which were analyzed by one-way ANOVA.

It has been well documented that coregulators (including activators and inhibitors) are essential for R-SMADs/SMAD4 complex-mediated regulation of the expression of target genes in the nucleus. Thus, we focused on investigating whether the coregulators are involved in SMAD4-mediated positive feedback regulation of the upstream receptors in the following study. First, we explored the interaction between SMAD4 and the potential functional proteins by using online programs, such as the STRING v11.0 database. After analysis, several transcriptional coregulators, including c-JUN, CREB1, c-Fos, FOXO3, HIF-1α, p53, p300, and SP1, were identified as the SMAD4-interacted regulators (Figure 4A and Figure S5A). Additionally, the interactions between candidate coregulators mentioned above and the upstream receptor genes (*ACVR1B*, *BMPR2*, and *TGFBR2*) of SMAD4 were analyzed by the JASPAR and GCBI online databases. Interestingly, analysis results showed that the binding motifs of c-JUN, CREB1 (cAMP responsive element binding protein 1), and SP1 (SP1 transcription factor) were located near the validated SBEs within the promoter of *ACVR1B*, *BMPR2*, and *TGFBR2* (Figure 4B and Figure S5B–D), suggesting that c-JUN, CREB1, and SP1 may serve as coregulators during SMAD4-mediated feedback regulation process.

To address this, the physical interactions between SMAD4 and c-JUN, CREB1, and SP1 in porcine GCs were first detected. Immunoprecipitation (IP) was performed and showed that SMAD4 interacts with c-JUN, CREB1, and SP1 in porcine GCs (Figure 4C). Next, we examined the effects of three coregulators on the expression and promoter activity of *ACVR1B*, *BMPR2*, and *TGFBR2* in porcine GCs with the indicated treatment. Results from qRT-PCR and Western blotting assays showed that inhibition of these coregulators dramatically suppressed the expression of related receptors at both mRNA and protein levels in the absence or presence of SMAD4 stimulation (Figure 4D–I and Figure S6A–C). Additionally, luciferase activity assays were performed and showed that knockdown of coregulators could dramatically inhibit the promoter activity of *ACVR1B*, *BMPR2*, and *TGFBR2* which were induced by SMAD4 overexpression (Figure 4J). Notably, we also found that the transcription activities of SMAD4 upstream receptors were significantly suppressed when the binding motifs of c-JUN, CREB1, and SP1 were mutated, even in the conditions of SMAD4 stimulation (Figure 4K). Together, our findings indicate that c-JUN, CREB1, and SP1 are three coactivators and necessary for the SMAD4-mediated feedback regulation of its upstream receptors, probably by forming regulatory complexes with SMAD4 in porcine GCs.

### 2.5. SMAD4 Interacts with Coactivators in Different Modes during Feedback Regulation Process

**Figure 5:**
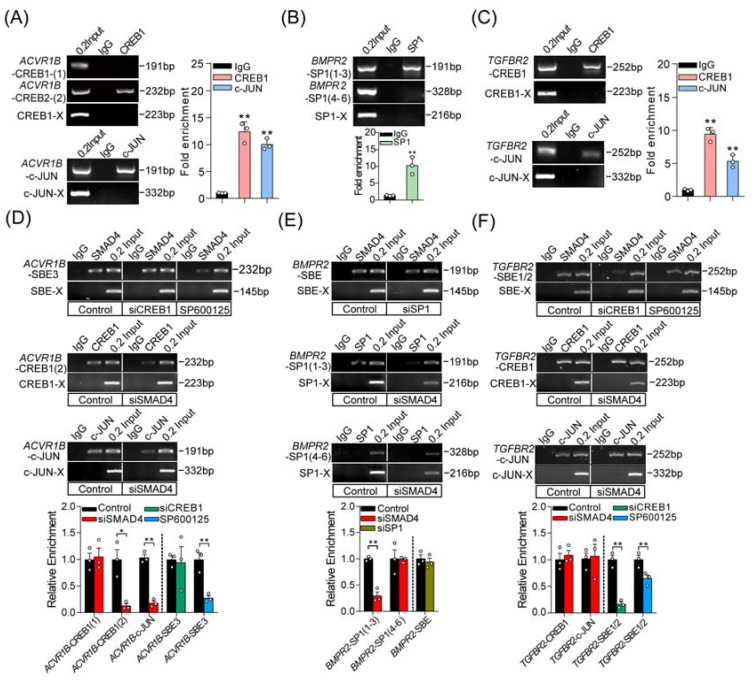
Three different interaction modes are identified between SMAD4 and coactivators in porcine GCs. (**A**–**C**) ChIP and ChIP-qPCR assays were performed to identify the interaction between coactivators (c-JUN, CREB1, and SP1) and the promoters of pig *ACVR1B* (**A**), *BMPR2* (**B**), and *TGFBR2* (**C**) (*n* = 3). (**D**–**F**) The interaction modes between SMAD4 and coactivators (c-JUN, CREB1, and SP1) during the SMAD4-mediated feedback regulation the transcription of *ACVR1B* (**D**), *BMPR2* (**E**), and *TGFBR2* (**F**) were identified by reciprocal ChIP and ChIP-qPCR assay (*n* = 3). Different treatments in porcine GCs were indicated in the boxes. Throughout, SBE-X, c-JUN-X, CREB1-X, and SP1-X indicate the negative control for ChIP assays with corresponding primary antibodies, respectively. Data are shown as the mean ± S.D. with three independent replicates (*n* = 3). The significance was analyzed by a two-tailed Student’s *t*-test, * *p* < 0.05, ** *p* < 0.01.

To further detect the interactions between coactivators and the promoters of pig *ACVR1B*, *BMPR2*, and *TGFBR2*, ChIP assays were performed and showed that c-JUN and CREB1 could interact with the promoters of *ACVR1B* and *TGFBR2*, while SP1 could interact with *TGFBR2* promoter by recognizing the corresponding response elements (Figure 5A–C). Based on the findings above, we next expected to explain whether SMAD4 interacts with the identified coactivators in the same or different modes in porcine GCs. To address this, reciprocal ChIP-qPCR assays were performed in the following study and suggested that three different interaction patterns existed between SMAD4 and coactivators during the SMAD4-mediated feedback regulation of upstream receptors, (i) the enrichment of CREB1 and c-JUN on the *ACVR1B* promoter were remarkably reduced after SMAD4 knockdown, while inactivation of c-JUN rather than CREB1 silencing impaired the SMAD4 enrichment on the *ACVR1B* promoter, indicating that SMAD4 may first form a complex with c-JUN, which further plays a positioning role in recruiting CREB1 to the *ACVR1B* promoter (Figure 5D); (ii) the enrichment of SP1 on the *BMPR2* promoter was dramatically reduced after SMAD4 silencing, while knockdown of SP1 had no effect on the enrichment of SMAD4 on the promoter of *BMPR2*, suggesting that SMAD4 plays an anchor and positioning role in recruiting SP1 to *BMPR2* promoter (Figure 5E); (iii) knockdown of SMAD4 had no effect on the enrichment of CREB1 and c-JUN on the promoter of *TGFBR2*, while the enrichment of SMAD4 was notably impaired after CREB1 silencing or c-JUN inhibition, which is in line with the fact that CREB1 and c-JUN may play positioning roles in recruiting SMAD4 to *TGFBR2* promoter (Figure 5F). Taken together, our findings revealed that SMAD4 acts as a transcription factor, and feedback induces the transcription of upstream receptors of the TGF-β family signaling pathways by interacting with three coactivators (CREB1, c-JUN, and SP1) in different modes.

## 3. Discussion

In this study, we attempted to investigate the biological functions of SMAD4 in mammalian ovarian GCs by screening for its target genes and interacting coregulators. Bioinformatics analyses indicate that SMAD4 is crucial for the normal states and functions of GCs, as well as oocyte development and maturation, which is consistent with previous studies [23,24]. Additionally, the differentially expressed genes were identified, and we noticed that the expressions of multiple members in the TGF-β family signaling pathways were influenced in GCs after knockdown of SMAD4, especially the three important receptors (ACVR1B, BMPR2, and TGFBR2) described in this study, which highlighted the feedback regulation activities of SMAD4 in mammal GCs. In the TGF-β family signaling pathways, SMAD4 has been considered the main feedback regulator that controls upstream ligands, receptors, and SMADs in different cell types, for instance, TGF-β1 in hepatic stellate cells [25], BMP2 in myoblasts [14], TGFBR1 in cardiac fibroblasts [6], BMPR2 in neuron [8], SMAD2 in C2C12 cells [9], and SMAD4 in Caco-2 cells [19]. In the present study, we showed that *ACVR1B*, *BMPR2*, and *TGFBR2* are three potential direct targets of SMAD4 and further indicated that SMAD4 can feedback activate the whole TGF-β family signaling pathways by inducing crucial receptors in a specific cell type: porcine GCs. Meanwhile, our findings have accumulated more evidence for feedback regulation in TGF-β family signaling pathways.

It has been well established that TGF-β and BMP signaling pathways are closely related, sharing multiple biological processes [26,27,28]. The crosstalk between the two pathways has been widely studied, and their members are well known to be regulated by each other [29,30]. It is worth noting that the core members belonging to TGF-β and BMP signaling pathways are quite different, except for SMAD4, the only common downstream molecule, which is crucial for TGF-β and BMP signal shuttling into the nucleus. Therefore, it is rare to identify the regulators that could mediate the downstream molecules simultaneously during feedback regulation of TGF-β and BMP signaling pathways in the same cell type and tissue or during the same biological process. A recent study has reported that endoglin, a newly identified common noncanonical receptor for TGF-β and BMP signaling pathways, could regulate the activities of both signaling pathways [11]. However, little is known regarding whether the core members within the pathways could feedback regulate their signaling. Our study suggests that SMAD4, the only common core component, may directly positive-feedback regulate both TGF-β and BMP signaling pathways in porcine GCs by acting as a TF and induce the transcription of its upstream receptors by interacting with the SBEs within their core promoter region. Notably, it is different from previous studies, which have shown that SMAD4 achieves feedback regulation of the TGF-β family signaling pathways via one or more mediators [6,7,31].

One of the highlights of this study is that we showed that SMAD4, the only Co-SMAD, has feedback activities to induce the expression of upstream receptors in GCs with a basal level of TGF-β ligands. As known, the ligands of TGF-β family signaling pathways, such as BMPs and TGF-βs, are secreted from GCs and further influence the activities of downstream signaling through both autocrine and paracrine methods [32,33]. Until now, few studies have reported the feedback activities of SMAD4 with exogenous ligands addition. In contrast, most previous studies involving SMAD4 were carried out in a basal level of ligands or without exogenous ligand addition, including ours and others [6,7], indicating that SMAD4 may function as a feedback regulator in GCs under the basal level of ligands or in a ligand-independent manner, which need further investigating. In addition to the expression of upstream receptors, we also demonstrated that SMAD4 induced the activities of R-SMADs by elevating their phosphorylation in porcine GCs, such as p-SMAD3 in our previous study [7] and p-SMAD1 in ongoing research (data not shown). Based on these findings, and taking into account that p-SMAD3 and p-SMAD1 are, respectively, activated by TGF-βs and BMPs, and further responsible for signal transduction [34], we hypothesize that the feedback activities of SMAD4 may be influenced or directly regulated by different kinds of TGF-β ligands, which is not fully understood and need further investigation. Another important issue that should be considered is whether the activities of I-SMADs (SMAD6/7) in GCs are regulated by SMAD4. It is generally believed that I-SMADs are activated depending on the phosphorylation of R-SMADs to prevent overactivation of the TGF-β signaling pathways [35,36]. According to this working model, we believe that the expression or activities of SMAD6/7 could be induced by SMAD4 in GCs, as the R-SMADs were feedback activated. Interestingly, we noticed that the transcription levels of SMAD6/7 are significantly upregulated in porcine GCs after knockdown of SMAD4, as shown in our RNA-seq data (Figure 1E), which is in line with the hypothesis.

**Figure 6:**
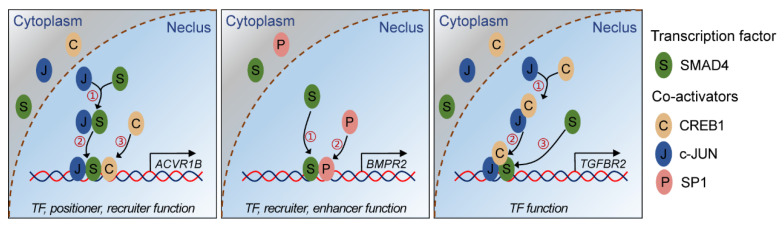
Schematic illustration showing the interaction modes between SMAD4 and coactivators during SMAD4-mediated feedback regulation of TGF-β family signaling pathway. S indicates transcription factor SMAD4, C, J, and P indicate the co-activators CREB1, c-JUN, and SP1, respectively.

In the nucleus, the R-SMADs/SMAD4 transcriptional complex regulates the spatial- and temporal-specific expression of target genes, usually along with other modulators and coregulators, such as TFs (e.g., β-catenin [11]) and transcriptional coregulators, including coactivators (e.g., p300/CBP [37]) and corepressors (e.g., Ski and SnoN [38]). However, the roles of SMAD4 in the interaction with these coregulators are quite different. For example, SMAD4 regulates the transcription of *SMIF*, *PAI-1*, *MMP2*, and *Rorc* by recruiting the p300/CBP complex, SNIP-1, and SKI, respectively [39]. During these processes mentioned above, SMAD4 only acts as a positioner and plays a protein-recruiter role. However, in other cases, SMAD4 functions as a recruited TF or directly binds to the promoter of target genes by specifically recognizing SBE motifs [40]. Based on the observations in this study, we propose a molecular mechanism model depicting the SMAD4-mediated feedback regulation of the TGF-β family signaling pathway (Figure 6). Briefly, SMAD4 acts as a TF and binds to the promoter of TGF-β family receptors (ACVR1B, BMPR2, and TGFBR2) by interacting with three coactivators (CREB1, c-JUN, and SP1) in different modes, which further elevates their expression levels in porcine GCs. Our findings demonstrate that coregulators are necessary for the SMAD4-mediated transcription of downstream target genes and also suggest that SMAD4 could regulate the target genes with different modes, even for those from the same family or signaling pathways.

Follicle-stimulating hormone (FSH), an essential gonadotropin, has been shown to be crucial for GC proliferation, folliculogenesis, and follicular development and maturation by activating FSHR signaling, which further regulates the downstream target gene expression, influences molecules activation, and interacts with other important signaling pathways [41]. It is worth noting that the expression and activities of the coactivators (c-JUN, CREB1, and SP1) detected in this study have been reported to be influenced by the FSH/FSHR signaling pathway in the mammalian reproductive system [42,43]. Additionally, the actions and functions of the TGF-β family signaling pathways in FSH-treated GCs have already been widely investigated, especially the deep interactions between SMAD4 and FSH [44,45]. For instance, Fortin et al. demonstrated that SMAD4 is essential for normal FSH synthesis and necessary for FSH-mediated female fertility [46]. In porcine GCs, it has been reported that knockdown of endogenous SMAD4 severely impairs the functions of FSH, such as cell proliferation and steroidogenesis [47]. Interestingly, our previous study reported that SMAD4 mediates the regulation of FSH to the normal states and functions of porcine GCs through miR-143/FSHR and miR-126*/FSHR axis [48,49,50], suggesting that SMAD4 not only forms complexes with the identified coactivators but may also induce their activities via FSH/FSHR signaling, which need investigating in our further research.

One issue will be important and necessary to resolve, but we still do not understand the precise manipulation mechanism of SMAD4 to influence the expression pattern (up- or downregulation) of target genes. As known, SMAD4 and R-SMADs function as TFs by recognizing the SMAD binding elements (SBEs) within the promoter region of target genes [19,51,52]. However, the SBE motifs are highly conserved among mammals, which we do not believe is the crucial factor leading to the different expression patterns of SMAD4-targeted genes. According to our findings in this study, we hypothesize that the coregulators that form transcriptional complexes with SMAD4 might be responsible for altering the expression pattern of SMAD4-targeted genes, but currently, we lack strong evidence for this hypothesis, which needs further investigation. Once it is resolved, the mechanisms of not only SMAD4 but also all TF-mediated differences in expression patterns of their target genes can be determined.

## 4. Materials and Methods

### 4.1. Materials

DMEM/F12 medium (#11320033), fetal bovine serum (FBS, #10100147), PBS (#10010072), Penicillin–streptomycin (#15140122), and Lipofectamine^®^ 3000 reagent (#L3000015) were obtained from Life Technologies Co. (Carlsbad, CA, USA). Dimethyl sulfoxide (DMSO, #2650), phenylmethylsulfonyl fluoride (PMSF, #P7626), 37% paraformaldehyde (#P6148), proteinase inhibitor (#P2714), glycine (#67419), horseradish peroxidase (HRP), and c-JUN inhibitor (SP600125, #S5567) were from Sigma-Aldrich (Darmstadt, Germany). Nonfat milk (#P0216), RIPA lysis buffer (#P0013B), RNase A (#ST578), RNase inhibitor (#R0102-2kU), and BCA Protein Assay Kit (#P0012S) were obtained from Beyotime Biotechnology (Shanghai, China). PVDF membrane (#3010040001) and Protein A/G magnetic beads (#LSKMAGT02) were purchased from Merck Millipore (Darmstadt, Germany). Primers used here were synthesized by TsingKe Biotechnology (Beijing, China), and the siRNAs used in this study were designed and synthesized by GenePharma (Shanghai, China). All chemicals and solutions were analytical reagent grade and all buffer components were endotoxin free or low endotoxin from Sigma-Aldrich, as available.

### 4.2. Animals

A total of 85 healthy, nonestrus, and sexually mature Duroc–Yorkshire–Landrace sows (*n* = 85; average mass = 110 kg, average age = 180 d) from Zhushun Biological Technology Co. (Nanjing, China) were randomly selected for bilateral ovary collection and in vitro GC culture. The sows were fed, looked after, and finally slaughtered for ovary collection according to the the Regulations for the Administration of Affairs Concerning Experimental Animals (No. 2 of the State Science and Technology Commission, 14 November 1988). All animal-related experiments in this study were approved, conducted, and supervised by the Animal Ethics Committee of Nanjing Agricultural University, China (SYXK (Su) 2017-0027, 22 June 2017).

### 4.3. Cell Culture and Treatment

Fresh porcine bilateral ovaries were collected and placed in a thermos flask with 37 °C PBS and transported back to the laboratory within 1 h. The collected ovaries were washed with 37 °C PBS five times, and porcine GCs were harvested from 2–5 mm nonatretic ovarian follicles by using a syringe with a 22-gauge needle. Porcine GCs were cultured in vitro, as previously described [23]. In brief, the isolated porcine GCs were washed with 37 °C PBS containing 1% penicillin–streptomycin (*v*/*v*) three times, and then seeded in cell culture plates with DEME/F12 medium containing 10% fetal bovine serum (FBS) with 1% penicillin–streptomycin in a 37 °C humid atmosphere with 5% CO_2_. After culture for 36 h, porcine GCs were washed with PBS twice to remove impurities and nonadherent cells (oocytes and GCs with poor states), and the medium was replaced with fresh medium for treatment preparation. All the cells used in this study were tested and found to be uncontaminated and mycoplasma-negative. For cell transfection, Lipofectamine^®^ 3000 transfection reagent was applied to transiently transfect the oligonucleotides and plasmids into the porcine GCs cultured in vitro according to the manufacturer’s instructions. For SP600125 (c-JUN inhibitor) treatment, the cell culture medium was replaced with fresh medium without FBS for 8 h, and then SP600125 was added into the medium with a final concentration of 75 and 150 μM.

### 4.4. RNA Isolation and Quantitative Real-Time PCR (qRT-PCR)

After treatment or transfection for 24 h, the total RNA from porcine GCs was isolated and purified by using TRIzol reagent (#15596026, ThermoFisher Scientific, Waltham, MA, USA). The quantity and quality of the purified total RNA were detected by NanoDrop 3000 spectrophotometer (Agilent Technologies, Santa Clara, CA, USA). The degradation and contamination of the total RNA sample were estimated by running on 1.0% agarose gel. In addition, an Agilent 2100 bioanalyzer (Agilent Technologies, USA) was used to detect the integrity of each RNA sample. For qRT-PCR assays, 1 μg of purified total RNA was reverse-transcribed into cDNA by using HiScript^®^ II Q-RT SuperMix (#R223-01, Vazyme Biotech Co., Ltd., Nanjing, China), according to the manufacturer’s instructions. Then, qRT-PCR was performed as described in our previous study [53], and the relative expression levels of interested genes were calculated through the 2^−ΔΔCt^ approach. Data from qRT-PCR assays were normalized to the expression level of glyceraldehyde-3-phosphate dehydrogenase (*GAPDH*). Each group contains at least three samples, and the experiments were performed with three independent replicates. The primers used for qRT-PCR are listed in Table S4.

### 4.5. Rapid Amplification of cDNA Ends (RACE)

The transcription start sites (TSS) of pig *ACVR1B*, *BMPR2*, and *TGFBR2* genes were obtained by using the Rapid Amplification of cDNA End (RACE) with SMARTer RACE 5′/3′ Kit (#634858, Clontech Laboratories, Inc., Santa Clara, CA, USA) according to the manufacturer’s instructions. Briefly, 4 μg of high-quality total RNA from porcine GCs was used for RACE-Ready cDNA synthesis, and the 5′-end of pig *ACVR1B*, *BMPR2*, and *TGFBR2* was amplified and identified with gene-specific primers. The gene-specific antisense primers designed for RACE assay are listed as follows: *ACVR1B*-GSP: CCAGGTCGAGAGAGGGCTCTGATGC; *BMPR2*-GSP: CCGACCCCGACGTGGAGAGGTCGT; *TGFBR2*-GSP: ATGGCCAGGTGCTCACTGAACTCCA. Then, PCR products were analyzed by electrophoresis on 2.0% agarose gel, and the clear DNA bands were collected and purified. Finally, the purified DNA fragments were cloned into a pClone007 vector, and the corresponding TSS of each gene was verified by Sanger sequencing.

### 4.6. Remapping on Pig Reference Genome and Data Reanalysis

To reanalyze the potential functional targets of SMAD4, the total clean tags obtained from our previous RNA-seq study were rechecked, and genome mapping was reperformed with the latest version background of the pig reference genome (*Sus Scrofa* RefSeq 11.1) by Top Hat v2.0. Then, the information of sequence data was converted into the gene expression level. For gene expression level normalization, reads per kilobase transcriptome per million mapped reads (RPKMs) method was used, and RPKM ≥ 1 was set as the threshold to determine the gene expression. The DEmRNAs were identified with the following cut-off criteria: (i) |−log_2_(fold change)| ≥ 0.59 (|fold change| ≥ 1.5), (ii) *p*-value ≤ 0.05, and (iii) FPKM ≥ 1 in all samples. To further evaluate the potential functions, roles, and biological processes of the DEmRNAs identified in this study, Kyoto Encyclopedia of Genes and Genomes (KEGG) and Gene Ontology (GO) were performed by using DAVID v6.8. The GO and KEGG terms with *p* ≤ 0.05 were considered as significant functional terms. For SMAD4-mediated gene–function and protein–protein interaction (PPI) network construction, the interactions among the function-known DEmRNAs were analyzed by the STRING v11.0 database (https://string-db.org/; accessed on 11 September 2020) with the basic settings interaction degree ≥ 1 and minimum required interaction score ≥ 0.9 [0, 1], which were further visualized using Cytoscape v3.7.2 software.

### 4.7. Bioinformatics Analysis

The potential promoters of pig *ACVR1B*, *BMPR2*, and *TGFBR2* genes were predicted and analyzed using two online databases, Softberry (http://linux1.softberry.com/all.html; accessed on 28 June 2019) and PromoterScan (http://www-bimas.cit.nih.gov/molbio/proscan/; accessed on 28 June 2019), with the prediction score cut-off setting of >0.8 [0, 1]. The candidate transcription factors which potentially target the core promoters of pig *ACVR1B*, *BMPR2*, and *TGFBR2* and their corresponding binding motifs, were analyzed by JASPAR (http://jaspar.genereg.net/; accessed on 18 December 2019) and the GCBI online database (https://www.gcbi.com.cn/; accessed on 20 December 2019). The SMAD4-associated proteins (including transcription factors and coregulators) were referred and obtained from the STRING v11.0 database with a minimum required interaction score criterion of ≥0.8 [0, 1].

### 4.8. SiRNAs and Inhibitors

To inhibit the endogenous expression of *CREB1* and *SP1* in porcine GCs, three gene-specific small interfering RNAs (siRNAs) for each target gene were designed and synthesized by GenePharma (Shanghai, China). The siRNAs used in this study are listed in Table S5. For c-JUN inhibition, SP600125, a well-known c-JUN specific inhibitor, was applied, and the concentration of SP600125 used in this study (75 and 150 μM) was arranged according to the manufacturer’s instructions. The inhibitory efficiency of the siRNAs and inhibitor in porcine GCs were detected at both mRNA and protein levels.

### 4.9. Plasmids Construction and Luciferase Activity Assay

To identify the core promoter of pig *ACVR1B*, *BMPR2*, and *TGFBR2* genes, the different fragments of pig *ACVR1B*, *BMPR2*, and *TGFBR2* promoters were amplified and cloned into a pGL3-Basic reporter vector between *Kpn*I and *Xho*I. To further detect the effects of SMAD4 on the transcription activity of pig *ACVR1B*, *BMPR2*, and *TGFBR2*, their promoters containing the wild-type SMAD4 binding sites (SBEs) were amplified and cloned into the pGL3-Basic reporter vector between *Kpn*I and *Xho*I. Additionally, the SBEs mutant-type vectors were generated by using Trelief^TM^ SoSoo Cloning Kit (#TSV-S1, Beijing TsingKe Biotech Co., Ltd., Beijing, China) according to the manufacturer’s instructions with the wild-type plasmids as templates. To analyze the target sites of transcriptional coactivators (c-JUN, CREB1, and SP1) within the promoter of pig *ACVR1B*, *BMPR2*, and *TGFBR2* genes, their promoters containing the wild or mutant type of binding motifs were synthesized and inserted into the pGL3-Basic reporter vector between *Kpn*I and *Xho*I. All the recombination plasmids used in the present study were verified by Sanger sequencing.

For luciferase activity detection, porcine GCs were collected after treatment or transfection for 24 h, and a Dual-Luciferase^®^ Reporter Assay System (#E1910, Promega, Madison, WI, USA) was used to measure the firefly luciferase and Renilla luciferase activities following the kit’s manual. The relative luciferase activity of each sample was calculated as the activity of firefly luciferase relative to Renilla luciferase. Each group has three samples, and the experiments were performed with three independent replicates.

### 4.10. Western Blotting

After treatment for 48 h, porcine GCs were collected, and Western blotting assays were performed as previously described [54]. In brief, the total protein from porcine GCs was extracted and collected by 200 μL of ice-cold RIPA lysis buffer with 1% PMSF. The concentration of total protein samples was measured by the BCA method. An equal amount (~15 μg) of total protein was separated on 4–20% SDS-PAGE gel after electrophoresis with 140 V for 1 h and subsequently transferred into PVDF membrane (Merck Millipore, Germany) at 110 V for 1.5 h. After incubation with 5% nonfat milk for 1.5 h at room temperature, the membranes with separated proteins were incubated with the primary antibodies at 4 °C overnight, followed by incubation with the corresponding secondary antibodies at room temperature for 1 h. The protein blots were visualized after incubation with ECL reagent (#E412-01, Vazyme Biotech Co., Ltd., Nanjing, China). The primary antibodies used here are listed below: anti-SMAD4 (1:1000, #10231-1-AP, ProteinTech, Nanjing, China), anti-ACVR1B (1:1000, #D120045, Sangon Biotech, Shanghai, China), anti-BMPR2 (1:1000, #D221406, Sangon Biotech, Shanghai, China), anti-TGFBR2 (1:800, #sc-400, Santa Cruz, USA), anti-CREB1 (1:1000, #9197, Cell Signaling Technology, Danvers, MA, USA), anti-c-JUN (1:2000, #9165, Cell Signaling Technology, USA), anti-SP1 (1:1000, #2250, Cell Signaling Technology, USA), and anti-GAPDH (1:3000, #TA802519, ORIGENE, Nanjing, China).

### 4.11. Immunoprecipitation (IP) and Chromatin Immunoprecipitation (ChIP)

To detect the interaction between SMAD4 and coregulators in porcine GCs, immunoprecipitation (IP) assays were performed. In brief, a total of 200 μL of protein extracted from porcine GCs were incubated with 4 μg of anti-SMAD4 antibody at 4 °C overnight to form the antibody/SMAD4-protein complex. Then, 15 μL of pretreated Protein A/G magnetic beads was added into the system and incubated with gentle mixing for 4 h at room temperature. Pretreatment of the magnetic beads was performed according to the manufacturer’s instructions. Subsequently, the Protein A/G magnetic beads with the anti-SMAD4 antibody/SMAD4-protein complex were pulled down, and the SMAD4-interacted proteins were isolated after elution, which were further identified by Western blotting assays. Antibody against IgG (Biogot, #BD0051) was used here as a negative control, and 100 μL of total untreated protein was applied as input.

To identify the binding motifs and enrichments of the transcription factors within the promoter of target genes, chromatin immunoprecipitation (ChIP) and ChIP-qPCR assays were conducted as previously described [48,55]. In brief, after transfection or treatment for 48 h, porcine GCs were harvested by RIPA lysis buffer with DNase inhibitor, and the protein/DNA complexes in the cells were crosslinked with 1% paraformaldehyde for 10 min. After that, 2.5 M glycine was added to quench at 37 °C and incubated for 10 min. Subsequently, the complexes were ultrasonic with the following settings: 40% output for 130 s (10 s on and 30 s off) at 4 °C, and then pulled down with corresponding antibodies. After decrosslinking, the enrichment of DNA fragments was analyzed by semiquantitative PCR or qPCR. Similar to IP, IgG was used as internal control for normalization of the specific antibody ChIP signals, and the unprocessed chromatin served as the input for fold enrichment from the same sample. The primers used for ChIP and ChIP-qPCR were listed in Table S6.

### 4.12. Statistical Analysis

Statistical analyses were performed by using GraphPad Prism v7.0 software (GraphPad software) and SPSS v20.0. All data were presented as the mean ± S.D. of three independent experiments. Comparison between two groups was performed by using a two-tailed Student’s *t*-test. Comparison among three or more different groups was conducted by using one-way ANOVA followed by S-N-K post hoc multiple comparisons. * *p* < 0.05 and ** *p* < 0.01 were considered statistically significant, and the significance levels are stated in the corresponding figure legends.

## 5. Conclusions

In summary, we reanalyzed the effects of SMAD4 knockdown on the transcriptome of porcine GCs and identified that three crucial TGF-β family signaling pathway upstream receptors (*ACVR1B*, *BMPR2*, and *TGFBR2*) were positive-feedback regulated by SMAD4 in porcine GCs. The findings suggest for the first time that SMAD4 forms transcriptional complexes with coactivators (c-JUN, CREB1, and SP1) in different interaction modes, which further feedback activates the TGF-β family signaling pathways by interacting with the promoters of its upstream receptors and induces their transcription in porcine GCs. Our results provide a theoretical basis and experimental evidence for the mechanism of SMAD4-mediated feedback regulation, which improves and expands the regulatory network, especially the feedback regulation modes of the TGF-β family signaling pathways in the ovary.

## Data Availability

The data presented in this study are available on request from the corresponding author.

## Supplementary Materials

The following are available online at https://www.mdpi.com/article/10.3390/ijms221810024/s1.

## Abbreviations

ChIP	chromatin immunoprecipitation
DEGs	differentially expressed genes
FBS	fetal bovine serum
FC	fold change
GC	granulosa cell
GO	Gene Ontology
KEGG	Kyoto Encyclopedia of Genes and Genomes
IP	immunoprecipitation
PPI	protein–protein interaction
RACE	rapid amplification of cDNA ends
RPKM	reads per kilobase transcriptome per million mapped reads
RNA-seq	high-throughput sequencing of RNA
SBE	SMAD4 binding element
siRNA	small interfering RNA
TF	transcription factor
TSS	transcription start site
