# Supplementary material for: SMAD4 Feedback Activates the Canonical TGF-β Family Signaling Pathways
Source: Int J Mol Sci. 2021 Sep 16;22(18):10024. doi: 10.3390/ijms221810024 (PMC8471547; doi:10.3390/ijms221810024)
Supplement: Supplementary file 1 [file ijms-22-10024-s001.zip › ijms-1333703-supplementary.pdf]

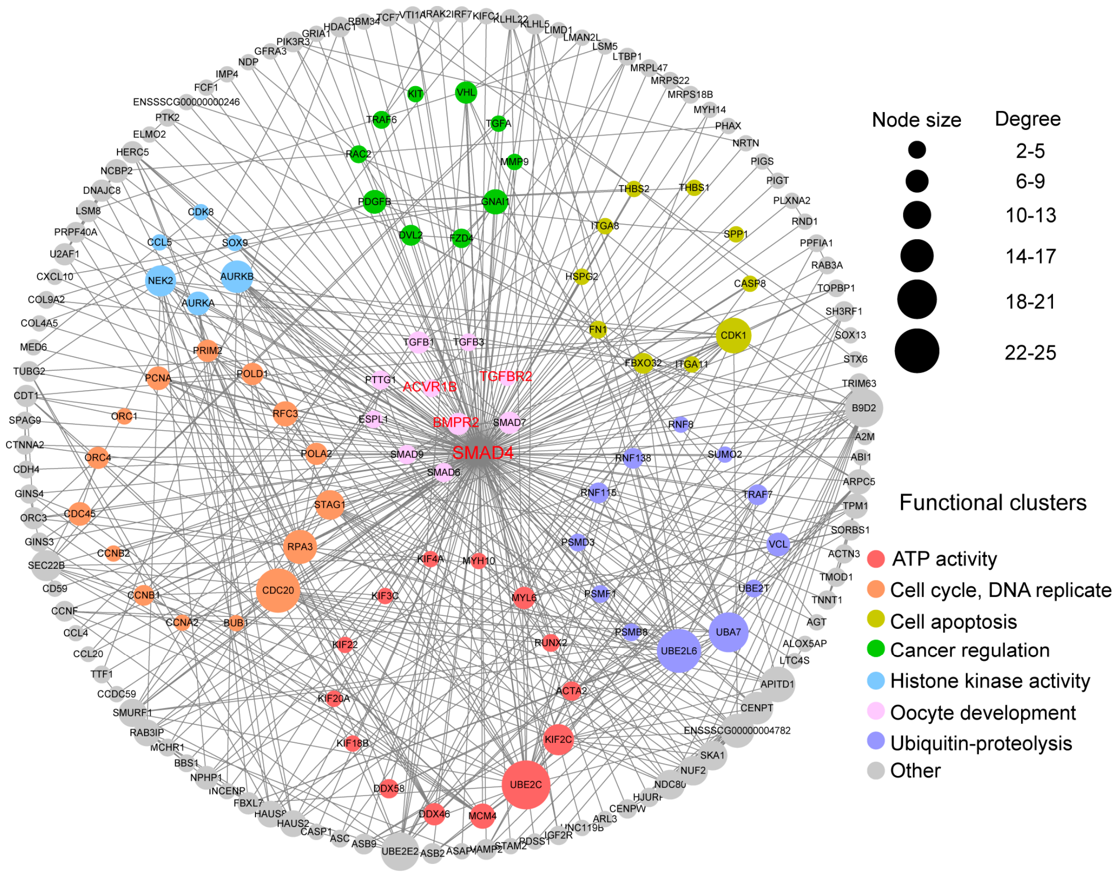

Figure S1. SMAD4-mediated gene-function interaction network. SMAD4-mediated DEmRNA-function interaction network was obtained by STRING database with the information of known connection data and visualized by Cytoscape 3.7.1. DEmRNAs are indicated as nodes, and their interactions are shown as edges. Nodes are grouped based on their known biological functions and their size reflects the interaction degree as indicated.

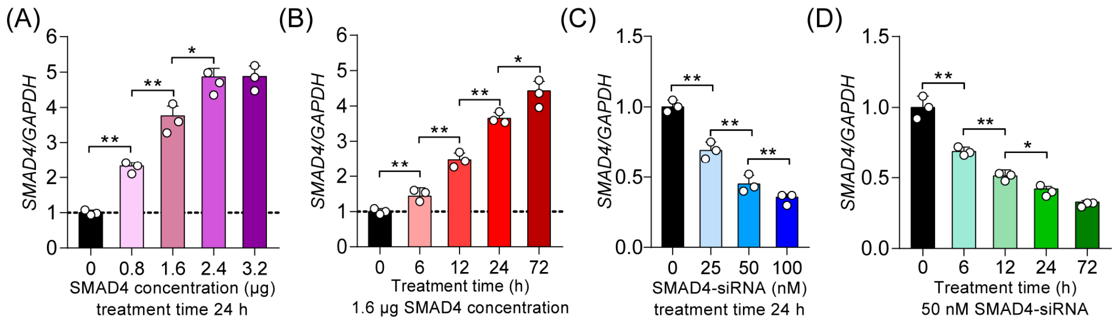

Figure S2. SMAD4 expression efficiency detection. (**A-D**) The mRNA levels of SMAD4 in porcine GCs treated as indicated were measured by qRT-PCR (*n*=3). Data were shown as mean ± S.D. with three independent replicates. *P*-values were calculated by a two-tailed Student's *t*-test. $^{*}P<0.05$, and $^{**}P<0.01$.

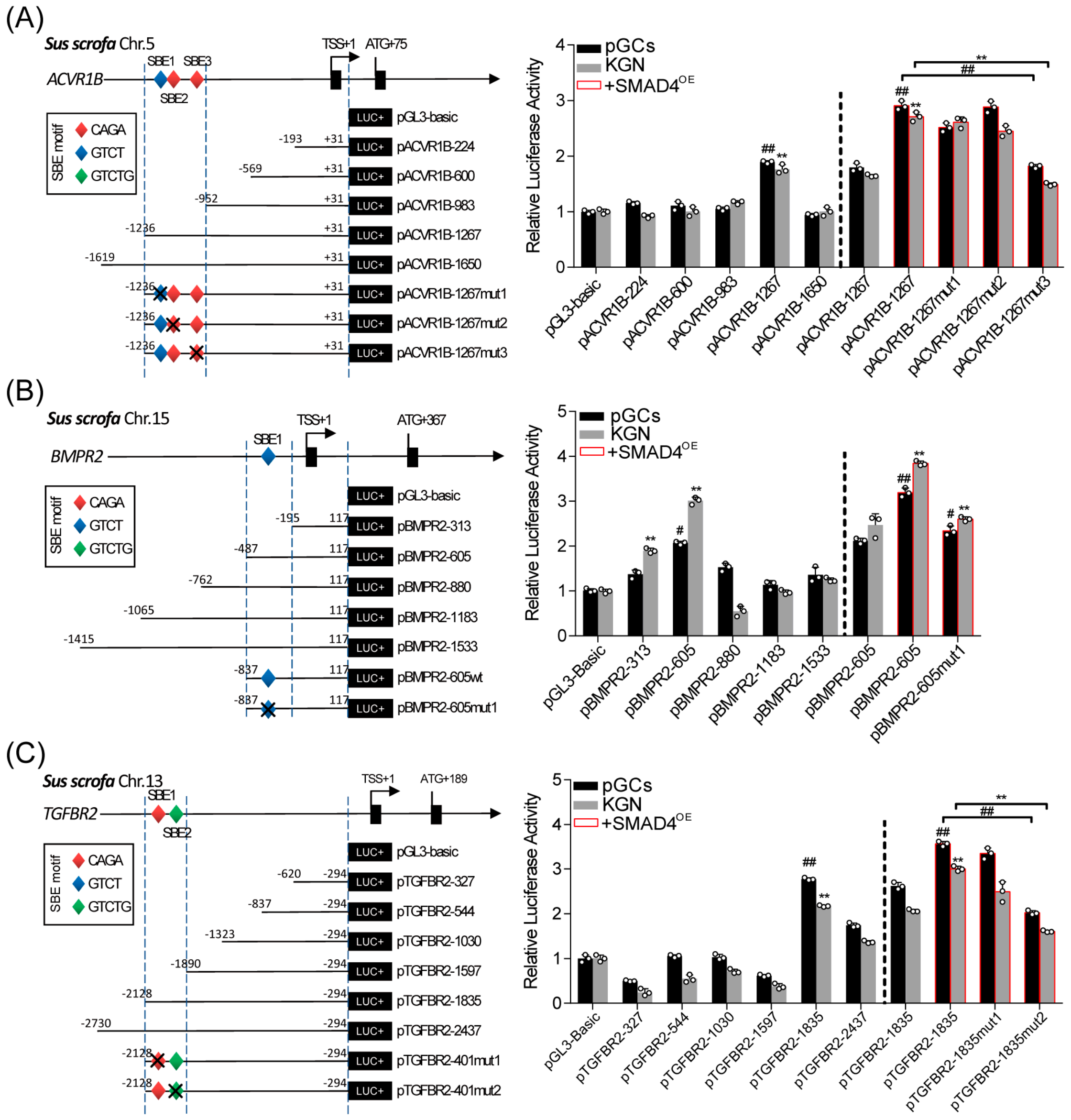

Figure S3. SMAD4 elevates the promoter activity of upstream receptors by binding to the SBEs within their promoters. (**A-C**). Left panel, diagrams showing the construction of recombined pGL3-basic reporter vectors containing the 5'-UTR of porcine *ACVR1B* (**A**), *BMPR2* (**B**), and *TGFBR2* (**C**) with wild-type or mutant type SBEs. The transcription start sites (TSSs) are indicated by black arrows. The different SBE motifs are shown as diamonds with different colors (blue: GTCT motif, red: CAGA motif, green: GTCTG motif). Right panel, Luciferase activity assays. The recombined reporter vectors were transfected into porcine GCs or KGN cells with or without SMAD4 stimulation for 24 h, and their activities were measured. Data were shown as mean ± S.D. with three independent repeats. $^{*,\#}$ $P < 0.05$ and $^{**,\#\#}$ $P < 0.01$ values were calculated by Student's *t*-test.

>Porcine *ACVR1B* core promoter (-1258/-889)

AATTAC**GTCT** AGATACTTAT ATCGCAA**CAG** **A**ACAAAGGGA GGGGAAAAAA TGTATACATG
-1250/-1247 -1229/-1226
TAAGTGTAAC TTGCTGTGCA GTGGGGAAAA AAAAAAACAA AACCTGCCCT TTACGTGATT

TCAGTCAAGC TCTTTTTCAA GCTAGGGAAA AGGAATTCTT TTTCAAAAAG ACATGGGTGA

TTCAGTTTAC CTTCTATAAG CAGTTTTTTC TAAATTTGAA AGTAACACCT GCTTATTGGT

AAAATATTTA GAAAACACTA AACTGTTTAA AGAAAAAGTA AAAAATTATT CTGGTGATAA

CTACTGATAA TACTGGGTCA TTTCCTTCCA GCCTTTTTTT CCAAA**CAGA**G GTAGAGTATC
-911/-908
AACAAAATTA

>Porcine *BMPR2* core promoter (-450/-195)

AAGAGAGGAA GAAGTACACC GGAGAGGGCT AGAAAGGGCA GCCTCCCCGC ACCTCCCCTC

CGCCCGCT**GT** **CT**TGGGGAGC TCGGGGCCCG GCCTGCAGAC CCCTCCCCTC CCCCGCTTCC
-381/-378
CACCGCCCCT CAGCCCTCCG GCTGGGACCT GCCAGACCCT CGCAGGATTG TTCCGCGAAG

ACGTGAAGGC GGTGGGTTTG TCCATGGAGG CAGGCACCTT TTTCGATCCA GTCGAGGAAG

AGGATTTGCT GTGCT

>Porcine *TGFBR2* core promoter (-2140/-1980)

TTGTTCATTT ATCCAACAAA TACCTATTAA ATATCTAGGT CCTGGGGATA CAGTAGTGAG

TGGAAGCTAA GATATCACTC CTGTCCTCAA GAAATCTGGA CTTGAGTCCA AAGAGAAGGG

AGAGGAAAGA ATGGCA**CAGA** ATTATTACAA AACTACAGCT GTAGTGGGCA GGCCGATGGT
-2004/-2001
GCCAAGGGAA TCTATAATAA AAT**GTCTG**CT GGGAGGGGAG GTCAGGGAGA GCTCACCCAT
-1936/-1932
CAAGGAAGTC

Figure S4. Characterization of the core promoter of porcine *ACVR1B*, *BMPR2*, and *TGFBR2*. The sequences showing the core promoter of porcine *ACVR1B*, *BMPR2*, and *TGFBR2*. The potential SBE motifs within these regions are labeled with different colors (blue: GTCT, red: CAGA, and green: GTCTG) and their locations were also shown below. TSS weas considered as +1.

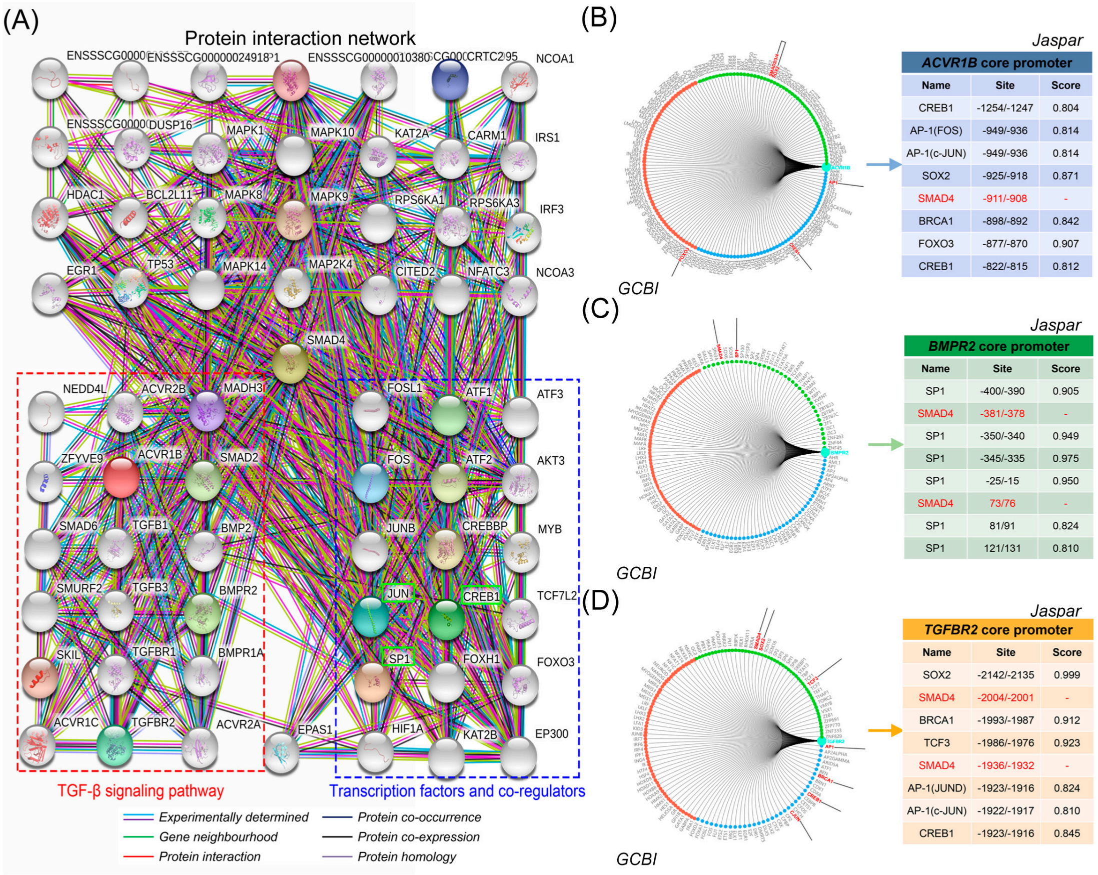

Figure S5. Predictive analysis of co-regulators involved in the SMAD4-mediated feedback regulation progress. (**A**) SMAD4-associated proteins were analyzed by STRING and their interactions were shown in a PPI interaction network. The red dotted box indicates a collection of TGF-β family members, and the blue dotted box shows a collection of transcription factors and co-regulators. Different interaction modes between nodes were indicated by colorful lines. (**B-D**)**.** Left panel, potential co-regulators target porcine *ACVR1B* (**B**), *BMPR2* (**C**), and *TGFBR2* (**D**) were predicted by GCBI database. Right panel, the binding motifs of co-regulators (c-JUN, CREB1, and SP1) and their locations within the core promoter of porcine *ACVR1B*, *BMPR2*, and *TGFBR2* were analyzed by JASPAR database. The locations of validated SBEs were shown in red. The score ranges from 0 (low) to 1 (high).

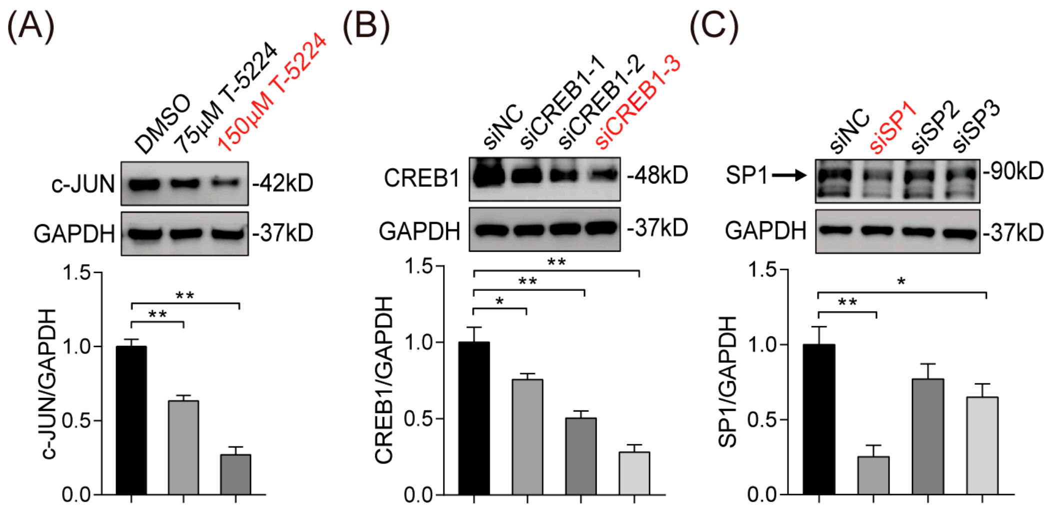

Figure S6. Knockdown or inhibitory efficiency detection. (**A**) The protein levels of c-JUN in porcine GCs treated with T-5224 at different concentration (75µM and 150µM) were detected by western blotting assay. (**B**) The protein levels of CREB1 in porcine GCs treated with three specific-siRNAs were measured by western blotting. (**C**) After treatment with three siRNAs specifically targeting SP1, the protein levels of SP1 were determined by western blotting assays. The concentration of T5224 and siRNAs labeled in red were chosen for further research. Data in **A-C** were described as mean ± S.D. with three independent repeats. *P*-values were calculated by a two-tailed Student's *t*-test. $^{*}P < 0.01$, $^{**}P < 0.01$.

Table S1. Differentially expressed mRNAs in SMAD4-reduced porcine GCs

Gene	Log_2FC	*P*-value	Regulation	Gene	Log_2FC	*P*-value	Regulation
MED29	6.476	3.23E-25	up	RFC3	0.612	4.34E-03	up
OASL	3.970	1.82E-13	up	APOO	0.611	3.99E-04	up
CEP57	3.861	2.91E-08	up	CKS2	0.610	7.97E-14	up
RSAD2	3.784	1.68E-22	up	PSMF1	0.609	2.86E-09	up
UNC119	3.668	1.21E-23	up	SPHK2	0.609	2.50E-03	up
IQCE	3.668	2.00E-04	up	ATF5	0.607	1.52E-06	up
KIF3C	3.561	6.39E-07	up	FKBP11	0.606	2.27E-09	up
UBN1	3.561	3.73E-04	up	RAB42	0.606	1.58E-04	up
ASB9	3.528	1.36E-22	up	MDK	0.605	1.05E-07	up
CTF1	3.320	9.80E-06	up	STAM2	0.604	3.93E-03	up
VPS41	3.200	3.17E-27	up	NCBP2	0.603	3.53E-04	up
RDM1	3.183	1.14E-03	up	NCAPH	0.601	1.27E-03	up
NDP	3.183	2.70E-06	up	CCT6A	0.600	1.80E-06	up
OAS1	3.157	1.63E-82	up	FGFR4	0.598	1.74E-04	up
CXCL10	3.031	3.24E-05	up	GLYCTK	0.596	2.00E-03	up
NPNT	3.031	6.34E-03	up	SERPINB2	0.595	7.27E-18	up
EIF2S2	2.963	2.89E-17	up	TMA16	0.595	1.05E-03	up
CAAT3	2.920	4.73E-03	up	POC1A	0.595	2.28E-06	up
SRSF12	2.861	9.64E-07	up	CCHCR1	0.595	1.24E-02	up
IFITM1	2.856	0.00E+00	up	CKS1B	0.594	2.69E-07	up
LRWD1	2.843	6.85E-08	up	NPHP1	0.593	3.75E-03	up
CSF1	2.800	2.43E-07	up	COPS2	0.593	4.06E-03	up
ZNF131	2.768	3.58E-12	up	C10orf54	0.586	3.22E-03	up
TEKT1	2.735	6.31E-04	up	ZNF205	0.586	4.13E-06	up
SOX9	2.709	1.10E-04	up	RUVBL2	0.585	1.95E-27	up
RSPH3	2.668	1.42E-05	up	EPN1	0.585	1.01E-15	up
MPHOSPH6	2.668	4.60E-04	up	KLF13	-0.586	1.06E-04	down
C9orf85	2.668	1.07E-02	up	MTURN	-0.586	1.01E-16	down
APRT	2.620	4.49E-11	up	FRZB	-0.586	3.31E-08	down
ANKRD26	2.561	1.44E-03	up	TUBG2	-0.587	1.69E-08	down
OPTC	2.446	1.87E-03	up	PPFIA1	-0.589	2.99E-03	down
ZNF467	2.446	6.47E-03	up	FITM2	-0.590	2.00E-08	down
TARS	2.430	4.60E-22	up	LRRC57	-0.590	3.08E-03	down
GSTP1	2.379	2.88E-11	up	CST3	-0.592	0.00E+00	down
NOC3L	2.364	1.82E-04	up	PIK3R3	-0.592	1.29E-02	down
TTC12	2.339	1.49E-08	up	HGSNAT	-0.592	4.78E-03	down
DHRS9	2.320	7.04E-03	up	CCDC92	-0.593	5.23E-04	down

CASP1	2.276	3.63E-03	up	SMAD7	-0.593	5.05E-06	down
EPS8	2.276	5.83E-03	up	SVEP1	-0.594	6.72E-11	down
STAG1	2.276	9.45E-03	up	KDSR	-0.595	2.35E-03	down
IFIT1	2.251	3.40E-10	up	LPPR3	-0.597	3.17E-03	down
PRICKLE4	2.223	3.82E-18	up	SLC25A13	-0.597	2.73E-09	down
BBS1	2.219	9.34E-03	up	IARS2	-0.597	3.20E-04	down
EIF3H	2.211	1.27E-09	up	UNC45B	-0.599	5.09E-28	down
TRAF7	2.210	1.07E-16	up	KCTD20	-0.600	4.16E-03	down
LSM8	2.203	1.97E-08	up	HOPX	-0.601	2.72E-03	down
NDC80	2.183	1.18E-04	up	LSS	-0.603	2.20E-09	down
SIVA	2.183	5.66E-04	up	COL16A1	-0.603	1.06E-07	down
RNF13	2.183	7.11E-03	up	ANO6	-0.603	1.39E-05	down
SA6L	2.183	2.32E-04	up	FN1	-0.603	2.03E-16	down
TUBA3D	2.141	1.69E-07	up	ANTXR1	-0.604	1.39E-07	down
CCL5	2.141	0.00E+00	up	RBM33	-0.604	7.48E-03	down
HINT3	2.124	2.65E-04	up	CD59	-0.604	6.55E-06	down
IFIT2	2.124	4.25E-08	up	EMC7	-0.607	5.81E-03	down
SIL1	2.115	3.02E-05	up	SLC45A3	-0.608	2.40E-04	down
IRF4	2.100	7.27E-06	up	UNC119B	-0.609	1.00E-11	down
BST2	2.094	0.00E+00	up	FBXO32	-0.609	1.75E-16	down
EVC2	2.083	4.92E-12	up	CYP2C91	-0.611	6.70E-03	down
BCL2A1	2.083	2.60E-03	up	TBC1D1	-0.611	2.79E-07	down
GCAT	2.063	2.11E-07	up	PDLIM1	-0.614	3.34E-16	down
MADD	2.049	1.79E-05	up	KREMEN1	-0.615	2.02E-03	down
LRRC16A	2.031	8.91E-04	up	INO80B	-0.617	2.06E-03	down
TSNAXIP1	2.031	1.04E-02	up	TRIO	-0.619	2.46E-05	down
HIST1H4D	2.031	2.93E-03	up	ARX	-0.619	7.76E-07	down
WNT7B	2.016	8.37E-06	up	TNNT1	-0.622	2.24E-04	down
FREM2	1.977	3.54E-04	up	TNFAIP8L3	-0.623	7.53E-16	down
SLC30A6	1.977	1.52E-07	up	C1orf226	-0.625	6.25E-03	down
TTC32	1.948	1.22E-02	up	VAMP2	-0.625	5.53E-04	down
VAMP5	1.947	5.96E-29	up	TXNRD1	-0.626	7.71E-04	down
LRRC56	1.931	1.31E-03	up	USP13	-0.627	6.69E-03	down
HLA-G	1.923	0.00E+00	up	PTPRM	-0.628	3.36E-06	down
MRPS16	1.901	5.68E-05	up	PAM	-0.629	3.77E-05	down
CKAP2	1.889	9.37E-03	up	STK24	-0.630	6.10E-03	down
S100A11	1.878	0.00E+00	up	EFCAB14	-0.632	4.89E-04	down
KIAA1328	1.861	2.39E-05	up	NNT	-0.632	5.35E-06	down
GSR	1.861	7.17E-04	up	KIAA0513	-0.633	2.22E-13	down
GBP4	1.845	3.08E-05	up	EMILIN1	-0.633	1.85E-12	down

IREB2	1.837	3.71E-03	up	VAT1L	-0.634	1.99E-04	down
ISG20	1.820	1.79E-06	up	CHI3L1	-0.635	1.47E-22	down
C12orf75	1.815	3.51E-20	up	UST	-0.636	3.25E-11	down
PGLYRP2	1.815	4.56E-03	up	EFNA1	-0.639	5.14E-03	down
EIF5A2	1.809	4.21E-03	up	SMURF1	-0.639	8.92E-04	down
PKMYT1	1.809	2.91E-03	up	LRRFIP1	-0.640	6.61E-07	down
FABP3	1.802	2.45E-08	up	RAB3A	-0.641	9.24E-03	down
PNPT1	1.800	4.18E-03	up	ABCC5	-0.642	3.35E-05	down
MLF1	1.787	6.31E-03	up	GAS6	-0.645	2.87E-14	down
TRMT5	1.775	1.38E-04	up	ADAM19	-0.645	5.43E-05	down
ZNF630	1.768	1.75E-03	up	GPR176	-0.645	7.85E-03	down
ALG6	1.754	4.81E-04	up	UBE2E2	-0.648	3.61E-04	down
DPF1	1.749	7.72E-03	up	GABARAP	-0.648	9.57E-07	down
CALR3	1.749	4.00E-04	up	FAM81A	-0.649	4.72E-03	down
AKR1E2	1.709	3.45E-05	up	LTBP1	-0.649	1.48E-18	down
SLA-5	1.708	0.00E+00	up	PTPRJ	-0.650	5.92E-03	down
DUS3L	1.704	3.49E-07	up	GDNF	-0.651	1.30E-02	down
ORC3	1.702	4.49E-05	up	THSD4	-0.652	1.20E-06	down
GRIA1	1.698	1.91E-06	up	PHF21A	-0.655	8.09E-03	down
BUB1B	1.650	2.11E-04	up	SIK2	-0.657	1.54E-04	down
COX11	1.650	3.46E-04	up	DST	-0.660	1.54E-05	down
SPINT1	1.650	1.10E-02	up	ARPC5	-0.660	8.50E-27	down
CTDNEP1	1.646	8.52E-18	up	HSPG2	-0.661	3.78E-19	down
CDK1	1.641	3.60E-07	up	LUZP1	-0.666	1.45E-07	down
UGGT2	1.639	6.19E-04	up	CHSY3	-0.667	3.46E-03	down
LGALS9	1.630	0.00E+00	up	TMEM2	-0.668	1.14E-06	down
MCHR1	1.627	9.09E-03	up	MRAS	-0.672	1.59E-11	down
AIPL1	1.616	9.69E-03	up	CTSL	-0.672	1.07E-19	down
ETV7	1.598	4.57E-04	up	RAB28	-0.674	1.87E-12	down
FAM126B	1.598	2.94E-03	up	COL4A5	-0.677	9.78E-06	down
TNF10-like	1.598	2.74E-03	up	MAMLD1	-0.680	6.48E-03	down
C2	1.595	1.39E-19	up	HIBADH	-0.681	1.26E-03	down
HLA-11a-like	1.592	0.00E+00	up	SUSD1	-0.681	1.22E-04	down
QRFPR	1.589	3.76E-04	up	TRAF5	-0.683	4.80E-03	down
MDFI	1.577	6.77E-04	up	COL8A1	-0.684	5.09E-09	down
GHDC	1.572	4.53E-05	up	EPB41L1	-0.685	1.51E-12	down
DDX58	1.571	4.96E-11	up	ACTA2	-0.686	0.00E+00	down
PAQR7	1.568	5.07E-08	up	ALOX12	-0.687	7.62E-04	down
HA-2a-like	1.568	3.20E-17	up	PTRHD1	-0.689	1.03E-03	down
SKA1	1.553	1.56E-03	up	CDV3	-0.690	1.76E-04	down

HMGB3	1.549	1.32E-05	up	FBXL7	-0.691	1.73E-04	down
CYP39A1	1.546	9.35E-05	up	EFTUD1	-0.691	5.14E-03	down
DTX3	1.539	8.89E-09	up	DAB2IP	-0.692	6.05E-03	down
TGFA	1.539	6.70E-05	up	PPT1	-0.698	7.81E-06	down
GZMA	1.539	7.11E-04	up	PCSK6	-0.698	1.17E-03	down
ERAP2	1.536	9.68E-04	up	ADAMDEC1	-0.699	2.74E-06	down
AP4S1	1.518	9.33E-04	up	HSDL1	-0.699	2.26E-03	down
KIF17	1.513	1.22E-02	up	GCDH	-0.700	2.96E-03	down
LY6G5B	1.513	8.31E-03	up	PRKCB	-0.700	9.63E-03	down
XRCC2	1.513	1.02E-03	up	AKAP2	-0.701	3.85E-10	down
SLA-1	1.503	0.00E+00	up	RPLP1	-0.702	2.29E-03	down
SP100	1.503	1.19E-04	up	CEBPB	-0.704	2.52E-06	down
AKAP7	1.493	1.16E-02	up	GADD45G	-0.705	5.32E-03	down
MX2	1.492	7.36E-28	up	CRIPT	-0.706	1.03E-03	down
CXCL5	1.486	3.32E-16	up	TRAF6	-0.706	2.78E-03	down
UQCC1	1.483	2.12E-03	up	FUCA1	-0.707	2.58E-22	down
IFI44L	1.480	2.03E-18	up	AHNAK	-0.708	2.11E-14	down
HERC5	1.465	2.56E-29	up	B4GALT5	-0.708	6.10E-06	down
PSMB9	1.455	3.01E-16	up	ASAP1	-0.710	1.77E-05	down
NHP2L1	1.446	6.68E-10	up	CAP1	-0.710	6.60E-18	down
xp_003846342	1.446	1.56E-04	up	FHL1	-0.711	2.06E-24	down
ZNF354A	1.446	4.18E-03	up	SPARC	-0.714	0.00E+00	down
NRTN	1.446	8.13E-04	up	ACVR1B	-0.714	1.34E-09	down
CMPK2	1.433	6.95E-24	up	PLA2G3	-0.721	1.45E-17	down
UBA7	1.426	2.12E-15	up	NAV2	-0.723	9.40E-03	down
ADSS-1	1.416	1.03E-04	up	LIMD1	-0.724	9.37E-04	down
PIFO	1.411	3.19E-05	up	COL9A2	-0.725	1.90E-05	down
JMJD7	1.402	3.87E-03	up	MYLK	-0.728	2.84E-06	down
PLAC8	1.391	2.25E-17	up	EDNRA	-0.730	4.57E-06	down
HDAC1	1.390	3.05E-20	up	TTC13	-0.730	3.81E-03	down
GORAB	1.385	9.25E-04	up	SH3RF1	-0.731	1.60E-05	down
PTTG1	1.374	9.37E-15	up	BPI	-0.731	1.26E-11	down
SLA-3	1.374	0.00E+00	up	CD34	-0.732	6.96E-08	down
RAC2	1.364	6.52E-03	up	VHL	-0.733	1.74E-03	down
SULT1C4	1.357	4.92E-05	up	CRIM1	-0.734	1.08E-22	down
EXOG	1.356	9.32E-03	up	CHRDL1	-0.735	2.08E-19	down
C1orf52	1.353	3.02E-04	up	PRR15	-0.737	2.95E-06	down
SLC35G3	1.346	3.17E-03	up	FRY	-0.738	3.87E-03	down
VRK1	1.325	8.94E-05	up	ITGA6	-0.739	8.03E-05	down
RNF115	1.320	4.76E-05	up	EPB41L3	-0.740	3.68E-03	down

IRAK2	1.320	6.62E-07	up	ATP6V1A	-0.741	1.78E-17	down
WASPH1-like	1.320	8.66E-03	up	NRG4	-0.743	3.91E-03	down
BLOC1S6	1.320	2.28E-07	up	G0S2	-0.746	3.67E-06	down
sestrin-2-like	1.317	2.05E-10	up	TPM1	-0.747	1.24E-28	down
GJD4	1.308	6.85E-03	up	ARHGAP10	-0.748	1.30E-03	down
TRAPPC8	1.286	5.50E-04	up	BMPR2	-0.751	2.19E-09	down
CTSS	1.276	2.06E-03	up	TTC17	-0.751	3.42E-04	down
IFITM2	1.274	0.00E+00	up	FAM13A	-0.753	6.69E-03	down
HLA-A	1.274	0.00E+00	up	CCDC167	-0.753	2.62E-12	down
IFI27	1.262	0.00E+00	up	DDAH1	-0.754	3.50E-08	down
ARHGEF39	1.262	2.53E-03	up	PHD23-like	-0.757	2.89E-04	down
KDM4B	1.258	1.34E-20	up	HERPUD2	-0.759	6.84E-04	down
KIF18B	1.258	3.58E-04	up	ACP2	-0.760	7.22E-04	down
IMP4	1.257	2.56E-21	up	PALM2	-0.761	9.05E-04	down
CD2AP	1.248	2.12E-03	up	PPP1R12B	-0.764	7.86E-17	down
TRPC6	1.247	3.54E-03	up	TANC1	-0.765	5.41E-04	down
FAM198B	1.246	4.08E-13	up	FBN1	-0.766	4.96E-28	down
TWIST2	1.240	1.03E-03	up	VTI1A	-0.767	8.83E-03	down
BUB1	1.229	1.42E-04	up	CTNNA2	-0.768	1.47E-04	down
MAPKBP1	1.224	1.59E-03	up	ID2	-0.774	3.82E-30	down
H1.4-like	1.224	1.14E-02	up	SMAD9	-0.775	4.77E-05	down
CDT1	1.223	1.16E-08	up	C1orf43	-0.776	5.87E-08	down
FAM83D	1.221	1.87E-03	up	SCHIP1	-0.776	2.36E-05	down
PBK	1.220	1.01E-03	up	ITGA8	-0.777	2.65E-22	down
WDR37	1.219	3.20E-17	up	THBS1	-0.778	0.00E+00	down
UBL7	1.216	4.28E-19	up	LUM	-0.779	1.12E-03	down
IL18BP	1.215	3.01E-03	up	FHL3	-0.779	7.48E-20	down
TTC37	1.215	9.23E-03	up	LTBP2	-0.779	2.19E-08	down
IFITM3	1.213	0.00E+00	up	ALDH1B1	-0.779	3.53E-06	down
ESPL1	1.209	3.11E-06	up	WNK2	-0.779	3.13E-03	down
RNF17	1.205	8.22E-03	up	ERG	-0.781	2.45E-04	down
KCNAB3	1.202	5.27E-04	up	TIMP3	-0.781	2.25E-03	down
ARMC8	1.200	2.96E-08	up	FZD4	-0.781	1.42E-10	down
SSSCA1	1.199	2.25E-21	up	ARHGEF7	-0.784	3.54E-03	down
CMZ-8-like	1.197	1.38E-08	up	GRTP1	-0.788	9.97E-03	down
SEC22B	1.194	2.10E-07	up	SLC26A2	-0.790	1.44E-06	down
TMX3	1.191	1.55E-03	up	ADAM12	-0.792	3.03E-25	down
AGRN	1.185	1.71E-11	up	C1orf115	-0.796	9.71E-17	down
USP32	1.183	2.83E-03	up	CDYL2	-0.801	4.21E-05	down
Np_001008925	1.183	8.29E-05	up	OLFML2B	-0.802	4.87E-09	down

MFAP2	1.183	3.01E-03	up	ANXA8	-0.803	1.08E-15	down
MXD3	1.183	1.62E-06	up	RAI14	-0.805	8.24E-05	down
UTP23	1.183	1.64E-03	up	UBL5	-0.808	4.03E-11	down
SUMO2	1.183	1.87E-03	up	DESI2	-0.809	5.19E-09	down
UNC93B1	1.172	3.56E-27	up	PRUNE2	-0.809	2.97E-04	down
RPP25	1.170	2.08E-12	up	CCNDBP1	-0.810	6.34E-09	down
SCNN1D	1.169	5.29E-05	up	ARSB	-0.811	1.94E-15	down
B2M	1.167	0.00E+00	up	TMEM158	-0.813	3.00E-05	down
CHD6	1.162	7.27E-04	up	NAV3	-0.815	1.17E-03	down
SLC15A3	1.162	2.01E-24	up	DNAJC8	-0.815	7.60E-06	down
NDUFA6	1.162	4.31E-15	up	EPHB6	-0.816	1.63E-03	down
TLL1	1.151	3.43E-03	up	ECE1	-0.819	5.29E-17	down
PTK2	1.146	2.83E-05	up	KLHL5	-0.819	6.84E-03	down
LRTOMT	1.141	2.16E-03	up	TSPAN2	-0.820	2.59E-03	down
LRRN4CL	1.141	9.00E-04	up	AD3MB2	-0.822	2.82E-03	down
ELP5	1.137	9.84E-11	up	RND1	-0.822	1.54E-08	down
PGAM2	1.132	2.41E-06	up	LYSMD4	-0.824	1.12E-03	down
APITD1	1.130	2.22E-05	up	KANK1	-0.825	4.77E-03	down
SLC26A6	1.127	8.49E-08	up	PROS1	-0.826	2.70E-06	down
INCENP	1.126	2.50E-07	up	CLCN7	-0.826	8.17E-05	down
IRF7	1.125	2.47E-25	up	WBSCR17	-0.827	3.10E-03	down
FAXC	1.124	1.20E-02	up	SMAD4	-0.828	5.46E-03	down
CCNB2	1.120	4.24E-07	up	ARMC10	-0.828	3.79E-04	down
IQGAP3	1.120	3.00E-07	up	DCLK2	-0.828	1.47E-03	down
R3HCC1L	1.117	8.64E-06	up	PRELP	-0.828	1.74E-08	down
GDPD1	1.109	8.29E-03	up	NEDD9	-0.828	2.39E-05	down
CCNA2	1.104	7.82E-08	up	DENND3	-0.831	7.35E-03	down
LYRM7	1.102	7.11E-03	up	KIDINS220	-0.831	9.26E-06	down
FAM195A	1.102	1.10E-06	up	THBS2	-0.832	2.17E-20	down
MICB	1.099	3.77E-09	up	RASL11B	-0.836	1.53E-05	down
IFIT3	1.092	1.56E-24	up	CSGALNACT1	-0.836	2.21E-04	down
NUF2	1.092	7.51E-03	up	VCL	-0.837	6.33E-08	down
CCNB1	1.092	9.86E-12	up	NF-KB	-0.837	6.92E-03	down
CXCL2	1.086	5.69E-09	up	PYCARD	-0.838	1.71E-03	down
UBE2C	1.084	3.38E-16	up	MATF	-0.839	4.54E-04	down
TMEM150A	1.083	5.79E-06	up	MAML2	-0.845	2.74E-03	down
UDP-like	1.083	1.12E-02	up	CTNND2	-0.847	5.34E-05	down
GTSE1	1.082	1.60E-06	up	TGFBI	-0.849	9.30E-06	down
FAM84A	1.079	1.24E-02	up	GFRA3	-0.851	1.33E-02	down
TMPRSS11F	1.078	4.00E-04	up	KIAA1324	-0.852	2.23E-03	down

BATF3	1.076	2.14E-05	up	CRISPLD2	-0.853	6.35E-03	down
E2F1	1.075	4.87E-13	up	A2M	-0.854	1.05E-05	down
SPAG9	1.070	2.33E-03	up	KLHDC9	-0.855	4.29E-03	down
DTNB	1.067	9.61E-06	up	ZNF516	-0.857	1.20E-02	down
DUSP15	1.065	3.40E-08	up	MYOF	-0.859	1.78E-12	down
IL34	1.063	1.76E-04	up	PRKCE	-0.860	8.01E-04	down
MRPS23	1.057	2.37E-08	up	PLXNA2	-0.861	4.04E-05	down
KIF4A	1.057	9.72E-03	up	MFSD4	-0.867	3.51E-05	down
ZFR	1.052	7.12E-06	up	TJAP1	-0.867	7.57E-04	down
DDX1	1.051	1.21E-08	up	GALC	-0.867	4.72E-03	down
RNF138	1.044	2.95E-04	up	KLHL22	-0.870	9.34E-03	down
HDAC9	1.038	1.26E-02	up	THYN1	-0.872	1.49E-07	down
B3GALNT2	1.031	1.15E-05	up	MR1	-0.874	1.78E-05	down
FAM127A	1.031	6.93E-13	up	TBC1D2B	-0.875	1.90E-06	down
TOMM40	1.031	1.24E-03	up	MLANA	-0.876	9.42E-07	down
OSBPL11	1.031	1.26E-02	up	TCF7	-0.876	6.87E-05	down
LOC100506562	1.022	1.81E-06	up	CCL11	-0.876	2.63E-03	down
CEP170	1.021	7.61E-03	up	CAMK1D	-0.876	6.05E-03	down
PLRG1	1.021	8.08E-04	up	ALOX5AP	-0.881	2.34E-03	down
OAS2	1.020	1.15E-16	up	COL12A1	-0.887	3.14E-15	down
HBA2	1.019	4.73E-06	up	ELOVL6	-0.891	1.66E-03	down
POLD1	1.015	6.99E-09	up	RASGRP3	-0.899	3.21E-07	down
SMKR1	1.013	3.89E-04	up	NADSYN1	-0.901	3.10E-06	down
C6orf211	1.009	1.35E-03	up	F2	-0.902	5.09E-03	down
CIR1	1.002	8.87E-05	up	RRNAD1	-0.908	3.31E-05	down
ZNF81	0.999	5.73E-03	up	PID1	-0.910	6.39E-19	down
RGMA	0.994	7.30E-04	up	MRVI1	-0.913	2.72E-06	down
TMEM52	0.989	2.60E-06	up	DPP4	-0.914	7.48E-04	down
BFSP1	0.987	2.20E-03	up	NREP	-0.926	2.14E-05	down
C10orf11	0.987	9.68E-05	up	ELMO2	-0.927	2.20E-05	down
TMEM18	0.985	9.54E-10	up	CSRP2	-0.933	2.32E-17	down
CCDC97	0.984	1.23E-02	up	TGFB3	-0.933	1.82E-06	down
KIF2C	0.983	2.68E-10	up	MRPS18B	-0.935	3.55E-07	down
HCST	0.983	4.00E-04	up	SLC25A43	-0.940	2.51E-04	down
CTXN1	0.981	4.42E-03	up	KLHL40	-0.942	4.56E-05	down
TMEM144	0.981	2.42E-04	up	TMEM255A	-0.942	1.01E-39	down
TBC1D23	0.980	4.50E-04	up	PTPN14	-0.946	9.94E-03	down
EDNRB	0.980	1.05E-18	up	SUN1	-0.946	5.24E-04	down
SRXN1	0.980	4.50E-12	up	NBEAL2	-0.948	1.58E-03	down
HTRA2	0.974	1.15E-13	up	ITGA11	-0.950	2.86E-27	down

NDUFAF2	0.972	2.84E-08	up	REEP6	-0.951	5.49E-05	down
SYBU	0.970	4.28E-03	up	KLF9	-0.953	2.40E-10	down
TOPBP1	0.970	1.14E-03	up	ADAMTS9	-0.955	2.46E-05	down
SLA-7	0.964	5.07E-05	up	VDAC3	-0.957	4.59E-13	down
CLPX	0.963	2.99E-03	up	RHOJ	-0.958	3.10E-03	down
UBE2L6	0.962	8.06E-18	up	DDX19B	-0.959	2.55E-04	down
CDCA3	0.953	1.72E-18	up	CYP4F22	-0.960	2.54E-03	down
TMEM38B	0.951	1.46E-03	up	SMAD6	-0.962	1.76E-06	down
EXPORTIN5	0.945	2.85E-25	up	SMOC1	-0.963	7.38E-03	down
SDHAF4	0.939	2.24E-03	up	EIF4EBP2	-0.972	1.39E-16	down
ILKAP	0.938	1.68E-05	up	CD58	-0.973	4.83E-04	down
INSIG2	0.935	4.61E-03	up	SRP14	-0.975	2.79E-09	down
DYDC2	0.935	3.33E-03	up	MYH10	-0.976	3.52E-19	down
GNB4	0.935	7.38E-03	up	KLHL38	-0.981	1.31E-02	down
XAF1	0.933	5.80E-29	up	CORO2B	-0.987	2.23E-07	down
BAI2	0.933	9.49E-03	up	CSNK1G1	-0.987	1.61E-03	down
PPP1R2	0.931	2.97E-08	up	ABHD12B	-0.993	7.17E-03	down
C1QL4	0.930	1.08E-04	up	KALRN	-0.994	5.79E-04	down
FCF1	0.925	1.26E-21	up	PLEKHM3	-0.997	2.92E-03	down
SHARPIN	0.924	5.81E-11	up	RASL11A	-0.997	1.44E-07	down
HSD3B1	0.922	0.00E+00	up	ENO3	-0.998	1.37E-22	down
RBM34	0.921	2.44E-04	up	MORC2	-1.000	8.06E-14	down
ITPKB	0.920	1.65E-05	up	AGXT2	-1.002	1.33E-08	down
SPP1	0.918	1.33E-22	up	TMOD1	-1.005	8.91E-06	down
IL15RA	0.917	2.65E-03	up	KIAA1143	-1.008	4.44E-03	down
KCNK2	0.915	5.93E-04	up	SAA1	-1.009	1.82E-22	down
NID1	0.905	1.98E-03	up	RBP1	-1.012	1.47E-11	down
NAP1L3	0.903	6.52E-03	up	PHKA2	-1.013	5.08E-08	down
SPAG5	0.898	7.08E-04	up	C20orf196	-1.013	1.13E-02	down
FAM131C	0.897	1.49E-04	up	COL11A1	-1.019	1.05E-15	down
TPX2	0.897	4.13E-04	up	CNTNAP1	-1.022	5.20E-03	down
CCNF	0.896	1.11E-08	up	TAF9	-1.023	4.32E-03	down
DUSP19	0.895	8.96E-03	up	COLQ	-1.027	6.84E-04	down
DGKZ	0.893	3.52E-03	up	HS6ST2	-1.028	2.76E-03	down
ORAI2	0.892	3.14E-03	up	NPY1R	-1.030	4.54E-05	down
CCDC58	0.889	9.67E-06	up	GEM	-1.031	7.31E-14	down
KIAA0556	0.888	5.36E-05	up	TRIM63	-1.033	3.91E-10	down
ZNF678	0.887	6.79E-03	up	CSAD	-1.037	2.80E-03	down
ORC1	0.886	9.85E-04	up	MAST4	-1.037	1.40E-03	down
MARCH7	0.882	9.09E-03	up	N-acetylgluco	-1.037	1.52E-03	down

PSME2	0.878	2.21E-14	up	RFTN1	-1.037	4.84E-03	down
PRPF40A	0.874	2.50E-03	up	ZNF365	-1.038	2.69E-03	down
IFI35	0.873	5.38E-08	up	Protein-93	-1.043	2.70E-06	down
AURKA	0.873	5.84E-12	up	AFF2	-1.044	8.77E-04	down
SFXN2	0.872	3.28E-03	up	CDKN2B	-1.049	4.23E-04	down
MYL6	0.862	0.00E+00	up	CASQ2	-1.052	1.87E-03	down
ASPH	0.861	3.20E-09	up	KIT	-1.055	2.84E-06	down
SLA-DQB1	0.861	9.50E-03	up	SORBS1	-1.058	5.41E-08	down
SYK-like	0.861	3.09E-03	up	EFR3B	-1.060	4.51E-07	down
KPNA3	0.861	1.09E-04	up	MYH14	-1.063	1.02E-02	down
MITD1	0.861	1.07E-02	up	ZSWIM1	-1.063	7.99E-03	down
EPSTI1	0.861	9.27E-06	up	HSD17B7	-1.066	2.01E-03	down
UHRF2	0.861	5.55E-04	up	RTN4IP1	-1.069	1.49E-03	down
DBI	0.861	4.84E-03	up	CHGB	-1.071	1.15E-04	down
OAZ3	0.861	2.85E-03	up	CLIC6	-1.072	3.87E-03	down
GUCA1B	0.861	2.42E-03	up	STX6	-1.073	2.33E-05	down
CENPT	0.850	2.32E-03	up	CCM2	-1.079	2.18E-16	down
USP35	0.848	3.50E-03	up	PDE4DIP	-1.082	2.42E-03	down
AURKB	0.843	4.31E-14	up	NRP2	-1.082	1.52E-06	down
ERGIC2	0.838	4.08E-07	up	MRPS21	-1.087	4.97E-05	down
FARSB	0.838	5.88E-09	up	CCRN4L	-1.087	2.23E-04	down
SEMA3C	0.834	5.90E-04	up	CDK8	-1.096	3.69E-06	down
ORC4	0.831	1.04E-03	up	CD209	-1.101	4.70E-03	down
APLF	0.824	1.20E-02	up	PPP2R5E	-1.101	5.53E-05	down
CCDC34	0.820	8.23E-03	up	PGAP2	-1.107	2.20E-03	down
GRPEL2	0.820	7.36E-07	up	PIGS	-1.110	7.39E-04	down
KLF16	0.816	2.48E-04	up	LDB3	-1.121	5.65E-04	down
PHAX	0.816	1.16E-04	up	EPT1	-1.139	7.64E-03	down
Mitochondrial	0.815	2.30E-17	up	TM9SF2	-1.139	2.29E-07	down
KCNAB2	0.814	4.97E-03	up	SMIM13	-1.139	9.55E-03	down
FKBP14	0.813	2.12E-04	up	ACTN3	-1.144	3.35E-05	down
TMEM87A	0.813	4.26E-04	up	CALN1	-1.156	1.15E-03	down
PRKAR1B	0.810	8.12E-03	up	CPSF4	-1.171	5.76E-07	down
NPM3	0.809	1.62E-24	up	SLIT3	-1.180	1.36E-03	down
LSM5	0.809	3.38E-04	up	RNF8	-1.185	2.98E-03	down
TONSL	0.805	4.45E-04	up	RN5-8S	-1.190	0.00E+00	down
TMEM177	0.804	2.12E-03	up	GRAMD3	-1.190	2.08E-04	down
CDCA4	0.803	2.65E-03	up	ABI1	-1.191	5.11E-03	down
ARHGEF25	0.803	3.09E-04	up	YLPM1	-1.198	5.32E-04	down
SDAD1	0.803	2.82E-04	up	DENND5B	-1.200	9.65E-03	down

CCL20	0.801	8.15E-16	up	H/ACA1-like	-1.213	7.84E-04	down
RCOR2	0.801	8.82E-03	up	CRISPLD1	-1.213	2.38E-06	down
GINS4	0.800	4.56E-04	up	UBL3	-1.224	3.72E-03	down
CDK14	0.800	6.90E-04	up	SIRPB1	-1.249	2.53E-04	down
FOXS1	0.798	1.08E-02	up	SELP	-1.261	3.89E-06	down
DHX58	0.797	1.22E-25	up	IGF2R	-1.269	2.72E-09	down
MX1	0.796	5.78E-15	up	FHIT	-1.276	1.06E-02	down
PTEN	0.795	5.55E-03	up	SLC25A23	-1.276	1.82E-05	down
ATXN3	0.793	8.67E-07	up	HSPD1	-1.288	9.73E-03	down
PRIM2	0.792	1.13E-06	up	DFNB31	-1.297	2.52E-03	down
TTF1	0.790	8.00E-03	up	ASB2	-1.313	6.13E-04	down
KIFC1	0.788	1.27E-03	up	CCDC40	-1.316	3.09E-03	down
CCDC153	0.787	5.17E-03	up	GLIPR2	-1.317	2.43E-17	down
KCTD13	0.783	5.11E-04	up	NDRG4	-1.336	1.09E-07	down
ZC3H3	0.782	1.22E-02	up	FAM227B	-1.341	5.95E-03	down
RAD21	0.780	2.22E-03	up	BTD	-1.345	5.78E-03	down
BL3-7-like	0.779	9.05E-03	up	IGFBP5	-1.347	3.03E-23	down
CDC20	0.778	1.11E-24	up	NOS1AP	-1.361	1.20E-03	down
BATF2	0.777	4.47E-03	up	HEPHL1	-1.376	2.58E-20	down
LMAN2L	0.777	5.21E-04	up	MMP9	-1.378	2.51E-08	down
NEK2	0.776	1.13E-03	up	CAPZA2	-1.380	8.93E-07	down
POMC	0.776	2.04E-03	up	SPG21	-1.382	2.49E-20	down
RHCG	0.776	1.48E-06	up	GPR133	-1.390	4.80E-03	down
UBE2D3	0.774	1.57E-12	up	IDI1	-1.399	3.44E-06	down
TMEM106A	0.772	5.48E-07	up	AHSA1	-1.408	4.95E-16	down
PPP3CA	0.770	3.54E-03	up	CYP4F3	-1.415	8.85E-13	down
PRRT3	0.766	5.25E-05	up	HDC	-1.417	7.08E-18	down
PSMD3	0.765	9.18E-09	up	DAPK1	-1.419	9.12E-04	down
MRP1	0.765	9.85E-03	up	RECK	-1.419	1.17E-02	down
EEF1E1	0.765	4.05E-05	up	PTGIR	-1.419	1.14E-03	down
RAB30	0.765	9.25E-04	up	ZNRD1	-1.439	3.71E-18	down
UBE2S	0.764	8.08E-03	up	KCTD3	-1.455	2.06E-10	down
UBE2T	0.763	3.81E-13	up	POPDC2	-1.470	9.37E-04	down
MKI67	0.761	8.08E-05	up	SLC6A6	-1.482	3.49E-08	down
FAM174A	0.761	7.89E-04	up	FBLN7	-1.491	2.02E-06	down
TGFB1	0.757	4.81E-10	up	Arylsulfatase	-1.500	1.02E-18	down
NUSAP1	0.756	1.98E-05	up	AGT	-1.512	1.43E-08	down
NFKBID	0.756	8.14E-03	up	FAM71F1	-1.527	1.96E-04	down
CDH24	0.755	3.23E-03	up	HIST1H2BD	-1.554	2.55E-05	down
DDX46	0.754	8.76E-03	up	SLC29A4	-1.554	1.16E-02	down

PHKA1	0.754	2.71E-04	up	SOX13	-1.554	1.24E-02	down
MRPS22	0.754	4.34E-07	up	RPE	-1.559	3.86E-07	down
FUOM	0.754	2.46E-06	up	SAG	-1.598	6.13E-03	down
ADAMTS5	0.753	2.35E-03	up	PELP1	-1.631	2.39E-07	down
POLA2	0.752	2.58E-09	up	PAFAH1B3	-1.644	3.35E-06	down
GPALPP1	0.752	1.56E-03	up	RPA3	-1.654	7.41E-03	down
KIF22	0.750	4.44E-08	up	DIABLO	-1.669	6.94E-22	down
AP3S1	0.746	1.30E-03	up	Sarcoglycan-L	-1.669	8.14E-06	down
SYNE3	0.741	1.05E-02	up	PLEKHG2	-1.673	5.80E-05	down
IPO11	0.739	5.95E-03	up	ZFYVE28	-1.673	7.82E-03	down
HAUS8	0.737	3.86E-03	up	NFYA	-1.677	1.21E-02	down
CDC45	0.734	2.65E-06	up	DVL2	-1.683	1.46E-04	down
KLF2	0.731	6.68E-04	up	TGFBR2	-1.690	2.31E-13	down
LCMT1	0.731	3.37E-12	up	RPL18	-1.693	0.00E+00	down
MED6	0.729	2.37E-06	up	CCDC30	-1.698	7.80E-03	down
GINS3	0.728	2.19E-03	up	CCL23	-1.724	2.68E-03	down
ASF1B	0.725	3.01E-06	up	R-spondin3-L	-1.724	9.63E-03	down
ADAMTS4	0.723	9.06E-03	up	PI4K2B	-1.724	2.44E-04	down
PRR5-ARHGAP8	0.718	5.99E-04	up	SLC25A35	-1.724	5.08E-03	down
UBAP1	0.718	3.22E-07	up	RUNX2	-1.737	2.13E-04	down
KIF20A	0.717	1.03E-10	up	CYP27A1	-1.775	5.69E-16	down
B9D2	0.716	7.56E-10	up	PDGFB	-1.779	1.28E-08	down
IFIT5	0.715	6.84E-04	up	FAT4	-1.791	2.54E-03	down
CGREF1	0.715	9.79E-09	up	UBR5-like	-1.802	1.58E-04	down
GBP1	0.713	1.73E-04	up	ITIH4	-1.802	8.85E-05	down
MTG1	0.710	3.82E-06	up	CTRB2	-1.837	5.65E-13	down
CCDC59	0.709	2.91E-05	up	TSC1	-1.855	2.45E-03	down
PPIC	0.709	2.80E-09	up	CELA2A	-1.859	3.10E-07	down
TBCK	0.709	7.98E-03	up	CD79A	-1.905	9.19E-03	down
MYBL2	0.703	1.15E-16	up	HBE1	-1.946	1.09E-02	down
BRIX1	0.702	4.84E-05	up	HLA-DQA1	-1.946	1.62E-03	down
SLC29A1	0.702	7.14E-07	up	SLC35G2	-1.969	3.11E-03	down
ARL3	0.701	6.65E-20	up	R3HDM2	-1.991	1.84E-09	down
PSMB8	0.699	1.12E-20	up	ATG2B	-1.991	2.58E-05	down
USP18	0.695	7.66E-12	up	FEZ1	-2.013	1.73E-03	down
PARP9	0.693	4.50E-04	up	RAET1E	-2.013	1.20E-02	down
SHROOM1	0.693	3.08E-04	up	ZMYND12	-2.065	9.36E-03	down
HAX1	0.690	2.33E-05	up	FAM188B	-2.109	4.38E-08	down
GNAI1	0.689	3.99E-03	up	CDH4	-2.125	9.19E-06	down
MACROD1	0.689	1.65E-06	up	ATG5	-2.139	6.26E-05	down

RUSF1	0.686	1.02E-03	up	CCL4	-2.139	8.01E-03	down
EMC10	0.685	1.24E-18	up	TFB2M	-2.139	7.31E-04	down
CENPW	0.682	2.34E-03	up	LTC4S	-2.139	2.17E-03	down
PLEKHG5	0.682	6.13E-03	up	PIGT	-2.176	2.03E-29	down
STAT5A	0.681	1.85E-07	up	CCDC148	-2.309	7.66E-03	down
AVEN	0.680	2.26E-03	up	GDF1	-2.309	8.64E-03	down
DNAL4	0.679	1.53E-05	up	CASP8	-2.332	7.72E-04	down
HMGB1	0.678	9.16E-15	up	RSU1	-2.347	1.66E-02	down
MRPS18A	0.673	4.32E-03	up	HCN1	-2.361	8.37E-05	down
NFRKB	0.673	5.75E-05	up	RAB3IP	-2.387	1.20E-02	down
APOL3	0.671	3.48E-09	up	ENY2	-2.418	1.21E-19	down
CRBN	0.668	1.59E-05	up	VAC14	-2.487	2.98E-26	down
UBR5	0.668	1.50E-04	up	MDH1	-2.521	1.41E-25	down
GOLM2	0.668	1.36E-03	up	PKLR	-2.554	4.18E-03	down
PAK4-like	0.664	7.08E-05	up	RYBP	-2.554	9.18E-11	down
LTA4H	0.663	2.24E-04	up	C3orf33	-2.554	9.96E-03	down
LRRC73	0.663	7.98E-04	up	CEP44	-2.598	1.63E-06	down
NAT1	0.662	1.91E-03	up	PDZ-like	-2.598	1.16E-07	down
GNL1	0.662	1.42E-20	up	SGCD	-2.598	1.04E-04	down
PDSS1	0.661	8.08E-03	up	ATXN7L3B	-2.613	9.83E-12	down
LANCL1	0.660	3.61E-05	up	WDR70	-2.624	2.41E-10	down
MCM4	0.660	4.23E-15	up	TCEAL4	-2.641	5.49E-03	down
MCTS1	0.656	1.46E-10	up	GPR98	-2.641	1.78E-04	down
PTPN12	0.655	6.17E-03	up	SPON1	-2.641	2.00E-04	down
BCS1L	0.655	6.94E-04	up	TMEM181	-2.702	1.45E-21	down
MTO1	0.653	3.63E-04	up	GATB	-2.724	1.22E-07	down
UQCRB	0.652	1.11E-17	up	FBXL2	-2.724	1.06E-02	down
PEX11G	0.651	2.46E-03	up	LRBA	-2.724	1.05E-02	down
VAX2	0.650	3.00E-05	up	MYOZ2	-2.724	8.28E-04	down
MRPL47	0.648	1.90E-03	up	SLIT-ROBO2	-2.724	7.46E-03	down
ADK	0.646	2.17E-03	up	AKAP5	-2.771	2.81E-03	down
IFI44	0.646	5.93E-09	up	FGT10-like	-2.861	8.10E-04	down
PUSL1	0.646	1.12E-03	up	POLR3B	-2.868	1.40E-17	down
EMP2	0.644	2.09E-03	up	FBXO33	-2.905	3.61E-03	down
RBIS	0.642	4.38E-06	up	TRIM5	-2.923	9.33E-14	down
SHMT2	0.635	1.59E-27	up	DOCK2	-2.946	2.46E-03	down
BIK	0.634	2.04E-04	up	TBX22	-2.946	7.44E-06	down
HNRNPH3	0.633	7.70E-03	up	NEK11	-3.046	3.25E-05	down
TRNT1	0.632	2.25E-03	up	RGN	-3.046	8.31E-03	down
HJURP	0.630	1.00E-02	up	PERP	-3.059	1.73E-03	down

NSMCE1	0.630	1.76E-03	up	CEMIP	-3.073	0.00E+00	down
N4BP2L2	0.630	2.77E-03	up	NOV	-3.093	1.19E-03	down
HAUS2	0.630	4.74E-03	up	USP40	-3.309	3.22E-03	down
ADSS	0.628	1.25E-03	up	ITGA9	-3.309	3.50E-04	down
TSC22D4	0.627	6.97E-04	up	FBXW2	-3.416	1.84E-20	down
DPM1	0.625	1.80E-06	up	RAD51C	-3.461	4.51E-05	down
IMPA2	0.624	6.26E-03	up	CUX1	-3.481	3.74E-22	down
C7orf50	0.621	1.15E-13	up	ST6GALNAC3	-3.724	1.91E-04	down
PCNA	0.620	3.13E-09	up	NEURL4	-3.763	5.66E-07	down
BCAS4	0.619	2.81E-24	up	TMEM192	-3.783	3.71E-09	down
U2AF1	0.618	2.17E-07	up	SLC11A1	-3.946	2.06E-09	down
FUT4	0.618	5.70E-03	up	TSPAN7	-4.046	1.42E-05	down
FAM64A	0.616	1.25E-05	up	PAF1	-4.209	3.87E-19	down
MMACHC	0.616	5.10E-03	up	CPB1	-4.226	3.28E-15	down
MFSD2A	0.613	3.17E-04	up	CPA1	-5.648	2.08E-28	down
USMG5	0.612	2.18E-12	up	CTRB1	-7.598	1.26E-23	down

Table S2. GO enrichment analysis of DEmRNAs after SMAD4 inhibition

Category	Term ID	GO Terms	Count	*P*-value[1]	Fold Enrichment
GO_BP	GO:0006955	Immune response	20	7.31E-04	2.382329519
GO_BP	GO:0008284	Regulation of cell proliferation	15	2.75E-02	1.891849912
GO_BP	GO:0006954	Inflammatory response	15	2.75E-02	1.891849912
GO_BP	GO:0043066	Regulation of apoptosis	15	3.88E-02	1.804979253
GO_BP	GO:0045893	Regulation of transcription	15	3.88E-02	1.804979253
GO_BP	GO:0008360	Regulation of cell shape	12	4.78E-03	2.695435685
GO_BP	GO:0051607	Defense response to virus	12	9.98E-03	2.439834025
GO_BP	GO:0006468	Protein phosphorylation	11	1.75E-03	3.283996008
GO_BP	GO:0070374	Positive regulation of ERK1 and ERK2 cascade	11	1.93E-03	3.242946058
GO_BP	GO:0010628	Positive regulation of gene expression	11	2.13E-02	2.295891015
GO_BP	GO:0007155	Cell adhesion	11	4.09E-02	2.05901337
GO_BP	GO:0030198	Extracellular matrix organization	10	1.10E-04	5.127187444
GO_BP	GO:0051301	Cell division	9	7.11E-04	4.516288514
GO_BP	GO:0060326	Cell chemotaxis	9	1.60E-03	4.005010569
GO_BP	GO:0016567	Protein ubiquitination	9	3.85E-02	2.332588573
GO_BP	GO:0030308	Negative regulation of cell growth	8	3.00E-02	2.657471802
GO_BP	GO:0045071	Negative regulation of viral genome replication	7	3.03E-04	7.178062421
GO_BP	GO:0070098	Chemokine-mediated signaling pathway	7	4.23E-03	4.462038802
GO_BP	GO:0007067	Mitotic nuclear division	7	8.97E-03	3.839428737
GO_BP	GO:0007010	Cytoskeleton organization	7	1.83E-02	3.301908714
GO_BP	GO:0051091	DNA binding transcription factor activity	7	2.00E-02	3.237165406
GO_BP	GO:0043547	Positive regulation of GTPase activity	7	2.80E-02	3.001735194
GO_BP	GO:0071347	Cellular response to interleukin-1	6	9.08E-03	4.564850756
GO_BP	GO:0007179	Positive regulation TGF-β receptor signaling pathway	6	1.51E-02	4.043153527
GO_BP	GO:0009615	Response to virus	6	1.89E-02	3.824604688
GO_BP	GO:0007059	Chromosome segregation	6	2.11E-02	3.723957196
GO_BP	GO:0071356	cellular response to tumor necrosis factor	6	2.84E-02	3.451472523
GO_BP	GO:0007018	Microtubule-based movement	6	4.04E-02	3.144674965
GO_BP	GO:0007015	Actin filament organization	6	4.38E-02	3.076312466
GO_BP	GO:0006270	DNA replication initiation	5	4.85E-03	6.936783012
GO_BP	GO:0048247	Lymphocyte chemotaxis	5	7.39E-03	6.206595326
GO_BP	GO:0034644	Cellular response to UV	5	1.26E-02	5.360241418
GO_BP	GO:0002548	Monocyte chemotaxis	5	1.72E-02	4.913554633
GO_BP	GO:0043491	Protein kinase B signaling	5	1.72E-02	4.913554633
GO_BP	GO:0007173	Epidermal growth factor receptor signaling pathway	5	2.57E-02	4.367604119
GO_BP	GO:0071346	Cellular response to interferon-gamma	5	2.90E-02	4.211618257
GO_BP	GO:0034446	Substrate adhesion-dependent cell spreading	5	2.90E-02	4.211618257
GO_BP	GO:0001503	Ossification	5	3.25E-02	4.066390041
GO_BP	GO:0002474	Antigen processing and presentation of peptide antigen	4	1.24E-02	7.861687414

GO_BP	GO:0030511	TGF-beta receptor signaling pathway	4	1.57E-02	7.256942228
GO_BP	GO:0000070	Mitotic sister chromatid segregation	4	1.93E-02	6.738589212
GO_BP	GO:0043392	Negative regulation of DNA binding	4	1.93E-02	6.738589212
GO_BP	GO:0090026	Positive regulation of monocyte chemotaxis	4	2.34E-02	6.289349931
GO_BP	GO:0060267	Positive regulation of respiratory burst	3	1.01E-02	17.68879668
GO_BP	GO:0032727	Positive regulation of interferon-alpha production	3	1.64E-02	14.15103734
GO_BP	GO:0000212	Meiotic spindle organization	3	2.39E-02	11.79253112
GO_BP	GO:0010642	Platelet-derived growth factor receptor pathway	3	2.39E-02	11.79253112
GO_BP	GO:0043304	Regulation of mast cell degranulation	3	2.39E-02	11.79253112
GO_BP	GO:0035457	Cellular response to interferon-alpha	3	2.39E-02	11.79253112
GO_BP	GO:0051382	Kinetochore assembly	3	3.26E-02	10.10788382
GO_BP	GO:0032967	Positive regulation of collagen biosynthetic process	3	3.26E-02	10.10788382
GO_BP	GO:0033627	Cell adhesion mediated by integrin	3	3.26E-02	10.10788382
GO_BP	GO:0019370	Leukotriene biosynthetic process	3	4.22E-02	8.84439834
GO_CC	GO:0005737	Cytoplasm	120	5.03E-04	1.334442509
GO_CC	GO:0005634	Nucleus	104	4.18E-02	1.183553733
GO_CC	GO:0005615	Extracellular space	52	5.95E-04	1.627774688
GO_CC	GO:0005739	Mitochondrion	42	1.59E-02	1.441485919
GO_CC	GO:0005576	Extracellular region	27	2.16E-02	1.582396354
GO_CC	GO:0005813	Centrosome	23	6.33E-04	2.239276231
GO_CC	GO:0009986	Cell surface	20	1.19E-02	1.856277031
GO_CC	GO:0005578	Proteinaceous extracellular matrix	16	8.85E-04	2.687679983
GO_CC	GO:0005911	Cell-cell junction	11	7.28E-03	2.704997509
GO_CC	GO:0030496	Midbody	10	1.62E-03	3.614115129
GO_CC	GO:0005856	Cytoskeleton	9	2.56E-02	2.52562869
GO_CC	GO:0000776	Kinetochore	8	7.45E-04	5.157439968
GO_CC	GO:0005876	Spindle microtubule	7	5.87E-04	6.422004575
GO_CC	GO:0072686	Mitotic spindle	6	3.34E-03	5.724758364
GO_CC	GO:0005871	Kinesin complex	6	2.02E-02	3.766288398
GO_CC	GO:0005604	Basement membrane	5	3.14E-02	4.112613767
GO_CC	GO:0019005	SCF ubiquitin ligase complex	5	3.90E-02	3.847283847
GO_CC	GO:0042612	MHC class I protein complex	4	9.35E-03	8.67387631
GO_MF	GO:0005524	ATP binding	54	3.09E-02	1.31310748
GO_MF	GO:0003682	Chromatin binding	19	8.06E-03	1.967052121
GO_MF	GO:0005525	GTP binding	19	4.01E-02	1.648644403
GO_MF	GO:0008201	Heparin binding	10	6.60E-03	2.941740334
GO_MF	GO:0008009	Protein kinase binding	7	2.43E-03	4.966349859
GO_MF	GO:0050840	Chemokine activity	6	6.49E-04	8.040756914
GO_MF	GO:0003725	Double-stranded RNA binding	6	2.37E-02	3.618340611
GO_MF	GO:0048020	CCR chemokine receptor binding	5	5.57E-03	6.700630762
GO_MF	GO:0019901	Extracellular matrix binding	5	1.17E-02	5.48233426
GO_MF	GO:0005518	Collagen binding	4	9.05E-03	8.771734815

GO_MF	GO:0001530	Lipopolysaccharide binding	4	2.21E-02	6.432605531
GO_MF	GO:0000400	Four-way junction DNA binding	4	2.21E-02	6.432605531

[1]GO terms with *P*-value < 0.05 were considered as significant functional processes of DEmRNAs.

Table S3. KEGG pathway analysis of DEmRNAs after SMAD4 inhibition

Term ID	KEGG Terms	Count	Percentage %	*P*-value[1]	Fold. Enrichment
ssc05200	Pathways in cancer	29	3.761348898	3.50E-03	1.768298843
ssc04068	FoxO signaling pathway	11	1.426718547	4.66E-02	1.936555435
ssc04145	Phagosome	12	1.556420233	4.09E-02	1.964871249
ssc05202	Transcriptional misregulation in cancer	13	1.68612192	2.83E-02	2.002574371
ssc04114	Oocyte meiosis	10	1.297016861	4.21E-02	2.148139057
ssc04512	ECM-receptor interaction	9	1.167315175	2.13E-02	2.60163508
ssc04623	Cytosolic DNA-sensing pathway	7	0.907911803	4.42E-02	2.686934591
ssc04115	p53 signaling pathway	9	1.167315175	1.10E-02	2.926839465
ssc03050	Proteasome	6	0.778210117	4.11E-02	3.121962096
ssc04350	TGF-beta signaling pathway	11	1.426718547	1.46E-03	3.344959388
ssc04612	Antigen processing and presentation	10	1.297016861	1.77E-03	3.5476842
ssc04110	Cell cycle	22	2.853437095	1.13E-08	4.47933692
ssc03030	DNA replication	7	0.907911803	3.23E-03	4.682943144

[1]*P*-value < 0.05 was considered as a cut-off criteria to identify significantly enriched pathways.

Table S4. The primers used for qRT-PCR in this study

**Gene name**	**Primer**	**Primer sequences (5'-3')**	**Product size (bp)**
ACVR1B	Forward	CATCCTTGGCTTCATTGCTG	125
	Reverse	CGATGGTCACCGTGTAGCG	
BMPR2	Forward	GATAGTTGCCTTATGCTTTGGAT	191
	Reverse	CAACTGGACGTTCATCTAAGGA	
TGFBR2	Forward	TGGCTCCTGAAGTCCTAGAGT	154
	Reverse	GAACCAAAGGGTGGCTCAT	
SMAD4	Forward	AGAGCGTCAACCGGGAGATG	250
	Reverse	CAGCCAGGAGAAATCAAACAGG	
c-JUN	Forward	CCTTCTACGACGATGCCCTCA	110
	Reverse	GTCAGCCAGGTTCAGGGTCAT	
SP1	Forward	AAGCAACGCCATTGTCAGG	385
	Reverse	ACCTGGATTCCCGAAGCAC	
CREB1	Forward	ATTGCCACATTAGCCCAGGTA	363
	Reverse	CTCTTCAATCCTTGGCACTCC	
GAPDH	Forward	GATGGTGAAGGTCGGAGTG	220
	Reverse	CGAAGTTGTCATGGATGACC	

Table S5. The siRNAs used in this study

siRNA		Sequence (5' to 3')	Source
NC-siRNA (siNC)	Sense	UUCUCCGAACGUGUCACGUTT	In this study
	Anti-sense	ACGUGACACGUUCGGAGAATT	
siSMAD4	Sense	CACCAGGAAUUGAUCUCUCAGGAUU	In this study
	Anti-sense	AAUCCUGAGAGAUCAAUUCCUGGUG	
siCREB1-1	Sense	GCCCAGCAACCAAGUUGUUTT	In this study
	Anti-sense	AACAACUUGGTTGCUGGGCTT	
siCREB1-2	Sense	GCAGACAGUUCAAGUCCAUTT	In this study
	Anti-sense	AUGGACUUGAACUGUCUGCTT	
siCREB1-3	Sense	GCAACAUCAUCUGCUCCCATT	In this study
	Anti-sense	UGGGAGCAGAUGAUGUUGCTT	
siSP1-1	Sense	GCAACAUCAUUGCUGCUAUTT	In this study
	Anti-sense	AUAGCAGCAAUGAUGUUGCTT	
siSP1-2	Sense	GGGAAACGCUUCACACGUUTT	In this study
	Anti-sense	AACGUGUGAAGCGUUUCCCTT	
siSP1-3	Sense	GCCAUCAGUUCUGCCAGUUTT	In this study
	Anti-sense	AACUGGCAGAACUGAUGGCTT	
